# FoxO3a as a Positive Prognostic Marker and a Therapeutic Target in Tamoxifen-Resistant Breast Cancer

**DOI:** 10.3390/cancers11121858

**Published:** 2019-11-25

**Authors:** Michele Pellegrino, Pietro Rizza, Ada Donà, Alessandra Nigro, Elena Ricci, Marco Fiorillo, Ida Perrotta, Marilena Lanzino, Cinzia Giordano, Daniela Bonofiglio, Rosalinda Bruno, Federica Sotgia, Michael P. Lisanti, Diego Sisci, Catia Morelli

**Affiliations:** 1 Department of Pharmacy, Health and Nutritional Sciences, University of Calabria, Rende, 87036 Cosenza, Italy; michele.pellegrino@unical.it (M.P.); pietrorizz@yahoo.it (P.R.); nigroale16@gmail.com (A.N.); riccielena91@gmail.com (E.R.); marilena.lanzino@unical.it (M.L.); cinzia.giordano@unical.it (C.G.); daniela.bonofiglio@unical.it (D.B.); rosalinda.bruno@unical.it (R.B.); 2 Department of Hematologic Malignancies Translational Science, Beckman Research Institute, City of Hope, Monrovia, CA 91016, USA; adona@coh.org; 3 Translational Medicine, School of Environment and Life Sciences, Biomedical Research Centre (BRC), University of Salford, Greater Manchester M5 4WT, UK; fiorillo.marco86@gmail.com (M.F.); fsotgia@gmail.com (F.S.); michaelp.lisanti@gmail.com (M.P.L.); 4 Department of Biology, Ecology and Earth Sciences, Centre for Microscopy and Microanalysis (CM2), Transmission Electron Microscopy Laboratory, University of Calabria, Rende, 87036 Cosenza, Italy; ida.perrotta@unical.it

**Keywords:** breast cancer, FoxO3a, growth factors signaling, tamoxifen resistance

## Abstract

*Background:* Resistance to endocrine treatments is a major clinical challenge in the management of estrogen receptor positive breast cancers. Although multiple mechanisms leading to endocrine resistance have been proposed, the poor outcome of this subgroup of patients demands additional studies. *Methods:* FoxO3a involvement in the acquisition and reversion of tamoxifen resistance was assessed *in vitro* in three parental ER+ breast cancer cells, MCF-7, T47D and ZR-75-1, in the deriving Tamoxifen resistant models (TamR) and in Tet-inducible TamR/FoxO3a stable cell lines, by growth curves, PLA, siRNA, RT-PCR, Western blot, Immunofluorescence, Transmission Electron Microscopy, TUNEL, cell cycle, proteomics analyses and animal models. FoxO3a clinical relevance was validated *in silico* by Kaplan–Meier survival curves. *Results:* Here, we show that tamoxifen resistant breast cancer cells (TamR) express low FoxO3a levels. The hyperactive growth factors signaling, characterizing these cells, leads to FoxO3a hyper-phosphorylation and subsequent proteasomal degradation. FoxO3a re-expression by using TamR tetracycline inducible cells or by treating TamR with the anticonvulsant lamotrigine (LTG), restored the sensitivity to the antiestrogen and strongly reduced tumor mass in TamR-derived mouse xenografts. Proteomics data unveiled novel potential mediators of FoxO3a anti-proliferative and pro-apoptotic activity, while the Kaplan–Meier analysis showed that FoxO3a is predictive of a positive response to tamoxifen therapy in Luminal A breast cancer patients. *Conclusions:* Altogether, our data indicate that FoxO3a is a key target to be exploited in endocrine-resistant tumors. In this context, LTG, being able to induce FoxO3a, might represent a valid candidate in combination therapy to prevent resistance to tamoxifen in patients at risk.

## 1. Introduction

Resistance to endocrine therapy is today one of the major clinical challenges in the treatment of breast cancer (BC) patients. A deeper understanding of the molecular events involved in resistance certainly will contribute to enhance clinical success. The anti-estrogen tamoxifen has been the main adjuvant molecule used in the treatment of estrogen receptor alpha positive (ER+) BC patients in the last 50 years [1,2]. Despite the fact that tamoxifen therapy has provided reduced risk of relapse and decreased mortality for millions of ER+ BC patients, many of these acquire resistance to the drug [3,4]. The acquisition of resistance may be ascribed to a loss or mutation of ER even if BC cells (BCCs) which lost sensitivity to anti-hormonal treatment, often retain a normal ER functionality [3].

In anti-estrogen sensitive BCCs, tamoxifen reduces cell growth by activating apoptosis [5] and/or autophagy [6]. Considering that many anti-estrogen resistant BC still respond to cytotoxic drugs by activating apoptosis [7], anti-estrogens should act upstream of the effector mechanisms inducing either apoptosis or autophagy.

In tamoxifen resistant BC, ER has been reported to interact with deregulated growth factor (GFs) pathways, facilitating the growth of resistant cells [8]. The enhancement of GF signaling, particularly HER2 and EGFR, results in the increase of ERK1/2 phosphorylation [9] and activation of phosphatidylinositol 3-kinase (PI3-K)/Akt [10,11,12], including PTEN down regulation [13]. Specifically, Akt seems to play an important role in maintaining tamoxifen resistance [14]. One of the downstream targets of Akt, currently attracting a great interest in hormone dependent BC [15] and whose role has only been speculated in endocrine resistance [16], is Forkhead box class O (FoxO)3a. FoxO3a belongs to a subfamily of winged-helix transcription factors, whose functions are negatively regulated by PI3K/Akt signaling [17] and the RAS-ERK pathway [18]. In the presence of GFs, FoxO3a undergoes phosphorylation by Akt and SGK and, through the chaperone proteins 14–3–3, is exported into the cytoplasm remaining in a steady-state [19]. In response to ERK-induced phosphorylation, FoxO3a is degraded via the ubiquitin–proteasome pathway [18]. In the absence of GFs, FoxO factors are mainly located within nuclei and regulate a set of target genes. FoxOs have been established as *bona fide* tumor suppressor genes [20] since they promote cell cycle arrest, apoptosis, DNA damage repair and the protection of cells from oxidative stress [15]. Increasing interest in FoxO3a is emerging in the oncologic research field since its inhibition is sufficient to make cancer cells resistant to numerous conventional and novel anticancer therapeutics [21]. In addition, FoxO3a may also be considered an important protective factor in ER+ BCCs [22,23] and a good prognostic factor in Luminal-like BC (ER+ cases) [24] where it directly correlates with biomarkers of good prognosis and with longer BC specific survival. In this context, here, we investigated, for the first time, the protective role of FoxO3a in the progression of ER+ BC from a sensitive to a resistant phenotype to tamoxifen treatment. Moreover, since we recently demonstrated that the antiepileptic drug (AED) lamotrigine (LTG), similarly to other AEDs [25,26,27], is able to inhibit BC growth by inducing FoxO3a expression [28], its potential use as adjuvant to tamoxifen therapy has been proposed.

## 2. Results

### 2.1. FoxO3a Is Downregulated in Tamoxifen Resistant (TamR) BCCs

**Figure 1:**
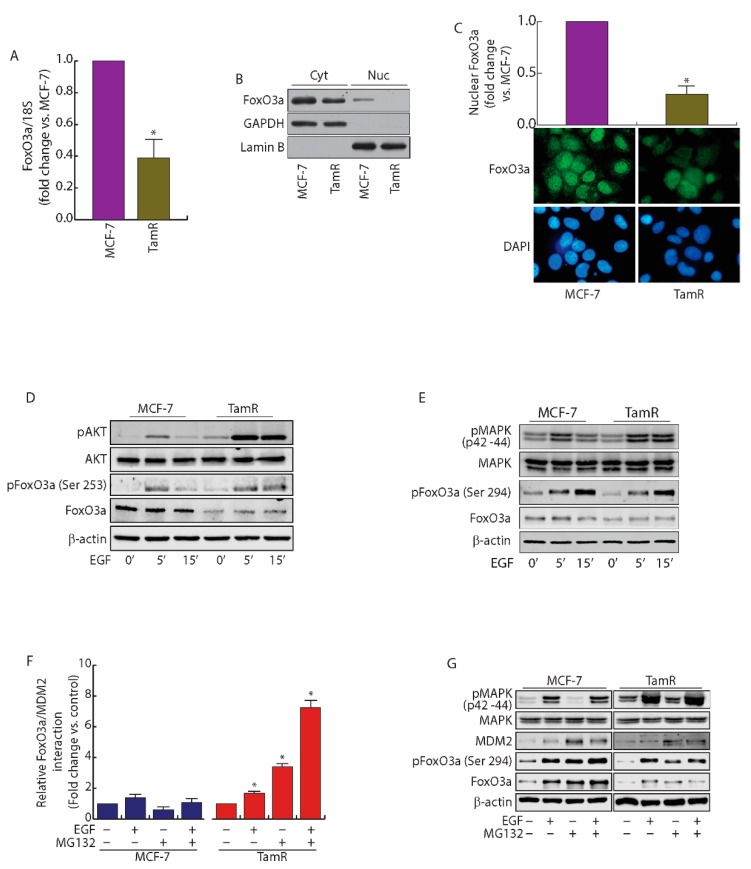
FoxO3a is downregulated in TamR BBCs. (**A**) FoxO3a transcripts were analyzed by real-time PCR in growing MCF-7 and TamR cells. Each sample was normalized vs. its 18S rRNA content and presented as fold enrichment versus MCF-7. The results represent the mean ± s.d. of three independent experiments. *, *p* < 0.01 vs. untreated. (**B**) Cytoplasmic and nuclear protein extracts from a duplicate set of cells were subjected to WB (30 μg/lane) to evaluate the subcellular localization of FoxO3a. GAPDH and Lamin B (cytosolic and nuclear markers, respectively) were used as loading controls and to assess the quality of the subcellular protein fractionation. (**C**) Immunostaining of FoxO3a expression and localization (green) in MCF-7 and TamR growing cells; nuclear integrity was visualized by DAPI (blue) (400x magnification) (**D**,**E**) Comparison between AKT (**D**) and MAPK (**E**) signal transduction pathways in MCF-7 and TamR cells. Cells were starved in PRF-SFM for 16 h and then treated or not with EGF (100 nM). Proteins were analyzed by WB, using the indicated antibodies. (**F**) PLA. MCF-7 and TamR cells were seeded in MW8 (Lab-Tek™ Chamber Slide System, Nunc™), left to adhere for 48 h, then starved in PRF-SFM and pre-treated with MG132 (20 μM) or left untreated (−). The next day, EGF (100 nM) was added for 30 min where indicated. Antibodies against FoxO3a and MDM2 were used to detect the active complexes. Captures of the stained FoxO3a/MDM2 complexes were analyzed by ImageJ software and their relative abundance vs. untreated samples was reported in the graph. The whole experiment, with the relative images, has been included in Supplementary Information (Figure S3). (**G**) A duplicate set of cells was treated as in (**F**) and protein expression was assessed by WB using indicated antibodies.

Considering the protective role of FoxO3a in ER+ BC, the potential involvement of FoxO3a in the acquisition of antiestrogen resistance was assessed in TamR cells, developed as described in Supplementary Information (Figure S1A,B). A significant decrease of both FoxO3a mRNA (Figure 1A) and protein expression, associated to a dramatic reduction of its nuclear localization (Figure 1B), was observed in TamR with respect to MCF-7 cells.

Since a 70 kDa isoform of FoxO3a has been recently described to translocate into the mitochondria of normal and cancer cells in response to metabolic stress stimuli, supporting mitochondrial metabolism and cell survival [29,30], FoxO3a expression was also evaluated in the isolated mitochondria of parental and resistant cells, in order to exclude the possibility that mitochondrial sequestration of FoxO3a in TamR could promote a resistant phenotype. Our results show that mitochondrial levels of FoxO3a are comparable in the two cell lines (Figure S1C).

Noteworthy, FoxO3a down-regulation is not related to the cell system, since it was also observed in tamoxifen resistant ZR-75-1/TR and T47D/TR cells (Figure S2A,D).

The loss of nuclear FoxO3a in TamR cells was also confirmed by immunostaining (Figure 1C). We supposed that FoxO3a downregulation in TamR cells could stem from the hyperactive GFs signaling observed in these cells, which results in PI3-K/Akt [10] and ERK1/2 increased activation [9]. Indeed, under EGF stimulation, both Akt and ERK1/2 were hyper-phosphorylated in TamR, compared to MCF-7 cells, resulting in FoxO3a sustained phosphorylation on the AKT target Ser253 residue and on the MAPK target Ser294 residue (Figure 1D,E) [21].

### 2.2. MDM2 Mediates FoxO3a Protein Degradation in TamR Cells

Since phosphorylation by both AKT and MAPK has been reported to trigger FoxO3a degradation through the SKP2-mediated [31] and/or MDM2-mediated ubiquitin-proteasome pathway [18], we hypothesized that hyperactive GFs pathways in TamR cells might cause FoxO3a degradation and its consequent decreased expression. The results obtained by investigating the interaction between FoxO3a and MDM2 by PLA confirm this hypothesis (Figure 1F and Figure S3). Interestingly, the highest number of PLA positive signals (~8 folds over control) was observed in TamR cells pre-treated with MG132 (an inhibitor of the 26S proteasome, used here to inhibit both FoxO3a [18] and MDM2 [32] proteasomal degradation, leading to their accumulation, as shown in Figure S4) and, then, exposed to EGF to induce MAPK-dependent FoxO3a phosphorylation (Figure 1G). Such a high degree of association between phosphorylated FoxO3a and MDM2 suggests that FoxO3a is preferentially targeted for MDM2-mediated ubiquitination in TamR cells. In fact, no significant increase of PLA signal in EGF treated MCF-7 cells, compared to untreated controls, even in MG132 treated samples, was observed (Figure 1F,G and Figure S3). 

### 2.3. Re-Expression of Foxo3a in Tamr Cells Restores the Sensitivity to the Antiestrogen

**Figure 2:**
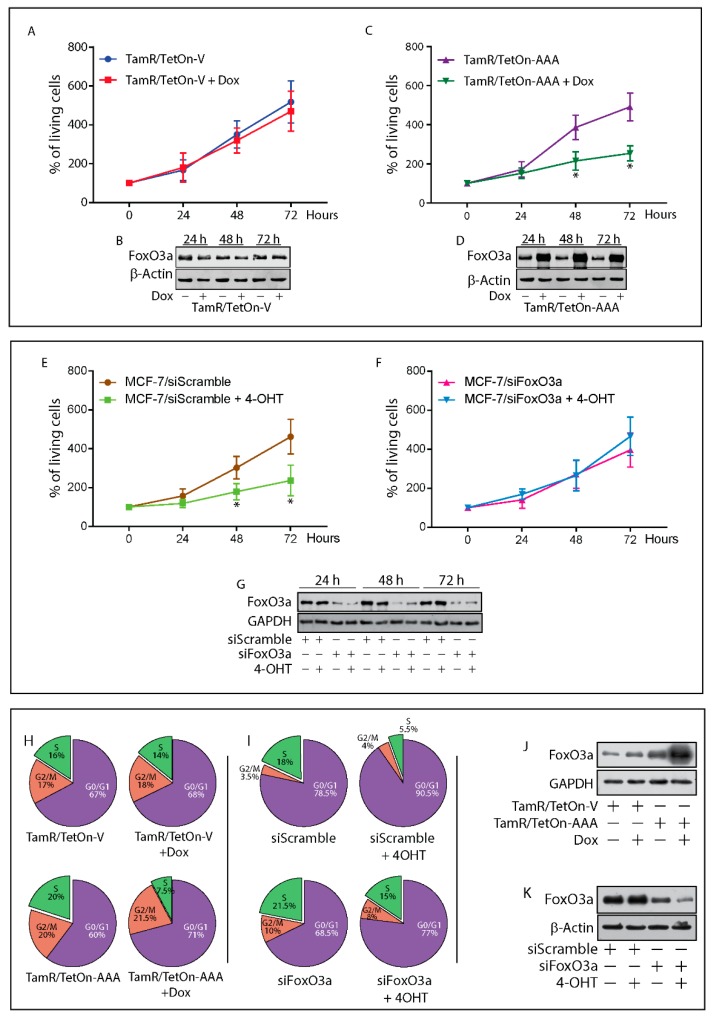

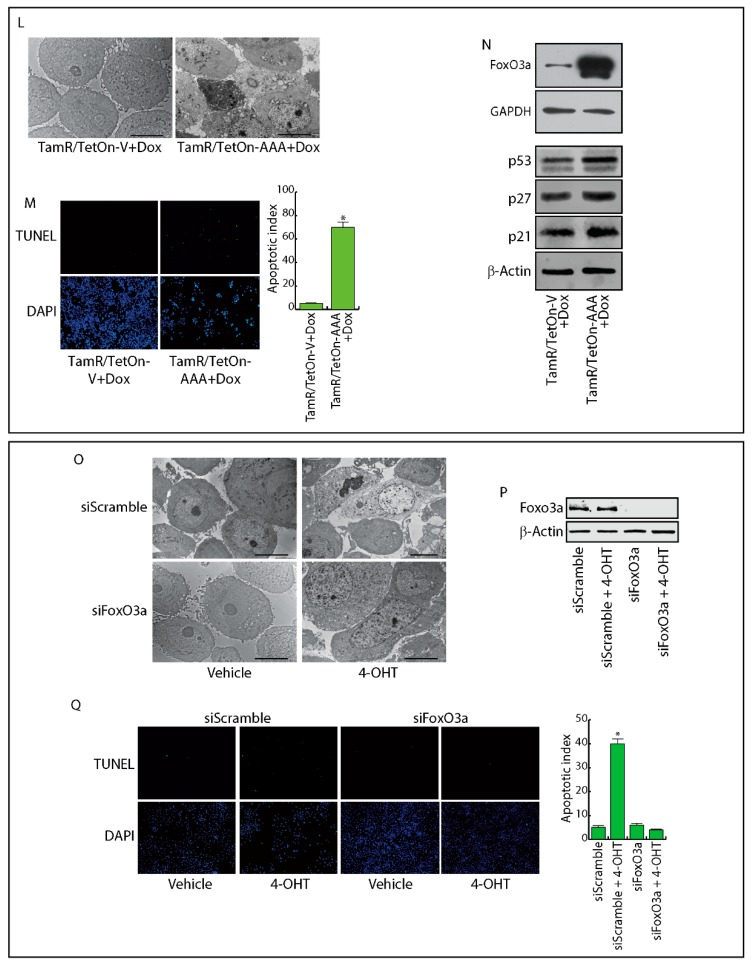
FoxO3a restores the sensitivity of tamoxifen resistant BCCs to tamoxifen. TamR/TetOn-V (**A**) and TamR/TetOn-AAA (**C**) cells were serum-starved for 24 h and then switched to 5% PRF-CT plus 1 µM 4-OHT and treated or not up to 72 h with Dox (3 µg/mL). MCF-7 cells were transfected with a Scramble siRNA (siScramble) (**E**) as control, or a siRNA for FoxO3a (siFoxO3a) (**F**). After 6 h, cells were serum-starved for 16 h and then shifted to 5% PRF-CT +/−4-OHT (1 µM) up to 72 h. 4-OHT treatment was renewed every day. Cells were then harvested by trypsinization and counted using trypan blue dye exclusion assay. Data represent the mean ± SD of three independent experiments. * *p* < 0.05 vs. relative untreated cells. The error bars indicate SD. Duplicate experiments were subjected to WB analysis to assess FoxO3a expression in Dox treated TamR/TetOn-V and TamR/TetOn-AAA cells (**B**,**D**), and in MCF-7 FoxO3a silenced cells (**G**). (**H**) TamR/TetOn-V and TamR/TetOn-AAA cells were treated as above described for 72 h and (**I**) siFoxO3a and siScramble MCF-7 cells, treated as above-described for 72 h were subjected to cell cycle analysis (see Materials and Methods). The percentage of cells in the G0/G1, S, and G2/M phases of the cell cycle are reported. The results are expressed as the mean from three independent experiments (Figure S5). A duplicate set of cells was subjected to WB to assess FoxO3a induction in Dox treated TamR/TetOn-AAA cells (**J**) and in MCF-7 FoxO3a silenced cells (**K**). TamR/TetOn-V and TamR/TetOn-AAA cells treated as before for 72 h and processed for TEM analysis (**L**) and TUNEL assay (**M**). siFoxO3a and siScramble MCF-7 cells were treated as before and subjected to TEM (**O**), and TUNEL analyses (**Q**). In TEM micrographs, scale bars: 10 µm. Original magnification: ×1200. In TUNEL assay, DAPI was used to counterstain the nuclei. Apoptotic cells were photographed at 10x magnification and then counted using Image J software. The graph on the right represents the corresponding apoptotic index (% apoptotic cells/total cell number in the field). Duplicate experiments were subjected to WB analysis to assess the expression of indicated proteins in Dox treated TamR/TetOn-V and TamR/TetOn-AAA cells (**N**) and in MCF-7 FoxO3a silenced cells (**P**). In WBsβ, Actin and GAPDH were used as loading controls.

To investigate whether FoxO3a re-expression could restore the sensitivity of TamR cells to the anti-estrogen treatment, we developed two Tet-On based tetracycline inducible TamR cell lines: TamR/TetOn-AAA, expressing the constitutively active triple mutant of FoxO3a (FoxO3aAAA) and the relative control TamR/TetOn-V, containing the empty vector. As expected, FoxO3aAAA induction by Doxycycline (Dox) inhibited cell growth and restored the sensitivity of TamR cells to 4-OHT (Figure 2A–D). Similar results were obtained in ZR-75-1/TR and T47D/TR cells (Figure S2B,C,D,E).

Interestingly, FoxO3a silencing (siFoxO3a), by reproducing FoxO3a status in TamR cells, was able to counteract 4-OHT induced growth arrest in MCF-7 cells (Figure 2E–G).

The cell cycle distribution of TamR/TetOn-AAA cells was analyzed after stimulation or not with Dox for 72 h. A significant inhibition of cell cycle progression was observed in FoxO3aAAA over-expressing cells compared to untreated cells, with a relevant increase of cells in the G1 phase and the concomitant decrease in the S-phase population. No relevant change in the G1 and S phase was observed in Dox treated TamR/TetOn-V cells, confirming that the effect is not due to Dox treatment, but to FoxO3a overexpression (Figure 2H,J).

Cell cycle distribution of MCF-7 cells silenced or not for FoxO3a, in the presence or absence of 4-OHT for 72 h, was able to counteract the antiestrogen effect, restoring the transition from G1 to S phase. As expected, 4-OHT caused a substantial increase in G1 phase of siScramble cells, lowering the S phase (Figure 2I,K).

### 2.4. FoxO3a Restores the Apoptotic Response to Tamoxifen in TamR Cells

In line with these results, Dox-induced over-expression of FoxO3aAAA was able to restore the normal sensitivity to the antiestrogen treatment by triggering the apoptotic pathway in TamR/TetOn-AAA cells (Figure 2L,N), while FoxO3a silencing in MCF-7 rescued the cells from 4-OHT dependent apoptosis (Figure 2O–Q). The ultrastructural effects of FoxO3aAAA over-expression in TamR/TetOn-AAA cells and those of FoxO3a silencing in MCF-7 cells have been assessed by TEM analysis. Most of the FoxO3aAAA over-expressing cells showed clear signs of injury (e.g., rarefaction of the nuclear chromatin and cytoplasm), as well as of apoptosis (Figure 2L, dark cell in the center), while 90% of Dox-treated TamR/TetOn-V cells appeared normal (Figure 2L). Interestingly, all the detached (dead) cells found in the supernatants of Dox-treated TamR/TetOn-AAA were apoptotic, confirming that FoxO3a induced death occurs through apoptosis (Figure S6A–D). These results were corroborated by TUNEL assay (Figure 2M) and by the increased expression of the pro-apoptotic p53, as well as of the cell cycle inhibitors p21 and p27, both well known FoxO3a target genes [33] (Figure 2N).

Conversely, FoxO3a silencing in 4-OHT-treated MCF-7 cells resulted in a significant reduction of cell damage (95% of intact cells) respect to 4-OHT-treated siScramble samples (75% of intact cells), maintaining the morphology of non-treated cells (siScramble and siFoxO3a samples, both showing ~90% of intact cells) with well-preserved cellular elements. A small percentage of apoptotic cells were only found in 4-OHT treated controls (Figure 2O). TUNEL assay gave comparable results (Figure 2Q).

### 2.5. Proteomic Analysis of TamR Cells Expressing Active FoxO3a: Impact on Proteins Controlling G1/S Transition, Apoptosis and GF Signals

**Figure 3:**
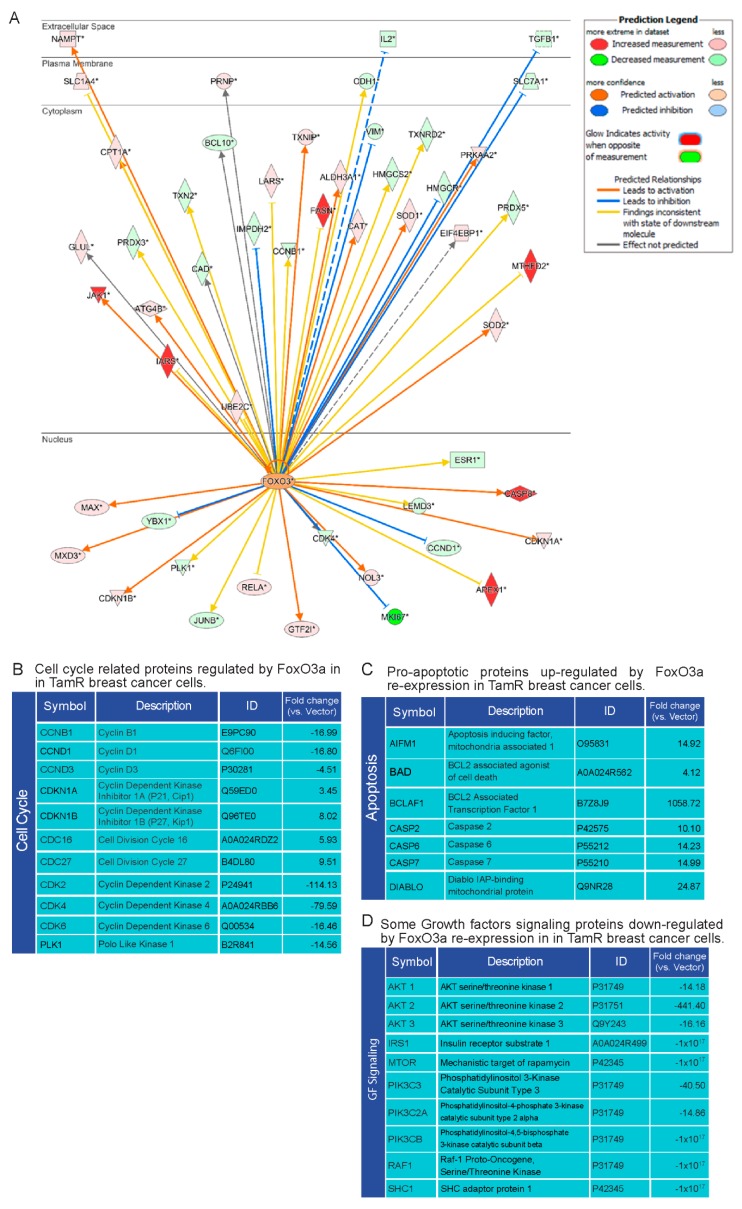
FoxO3a re-expression induces pro-apoptotic factors and down-regulates proteins involved in cell cycle regulation and GF signaling in TamR cells. (**A**) IPA reveals the direct FoxO3a downstream targets in TamR BC cells according to the Ingenuity Knowledge Database. The arrows indicate the predicted relationship, as reported in the upper left panel. The color intensity is related to the level of expression. (**B**–**D**) Proteins involved in the regulation of cell cycle (**B**), apoptosis (**C**) and GF signaling (**D**) that result modified of at least ±three-fold change by the active FoxO3a expression in TamR BC cells. For each protein, the symbol, the common name (Description), the data bank identification number (ID) and the fold-change expression in TamR/TetOn-AAA cells vs. TamR/TetOn-V cells are reported in the panels.

To analyze the molecular mechanisms through which FoxO3a re-expression can restore the response to the antiestrogen, Dox-treated TamR/TetOn-V and TamR/TetOn-AAA cells were subjected to unbiased proteomic analysis [34]. Ingenuity pathway analysis (IPA)) determined a large number of proteins whose expression resulted modified by active FoxO3a over-expression (Figure 3A). Several of the proteins regulated by FoxO3a over-expression are involved in cell cycle, apoptosis and GF signals. Thus, the block of cell cycle in G1/S phase in FoxO3aAAA over-expressing cells can presumably be due to the decrease of cell cycle modulators, such as Cyclin D1, Cyclin D3, CDK2, CDK4, CDK6, Cyclin B1 and PLK1. On the other hand, nuclear FoxO3a increases the expression of CDC16 and CDC27 and, as already shown, of p21^Cip1^, p27^Kip1^, all inhibitors of the G1/S transition (Figure 3B). Cell cycle perturbation by FoxO3aAAA was paralleled by an increase in the expression of pro-apoptotic factors such as AIFM1, BAD, BCLAF1, Caspase 2, 6 and 7, and DIABLO indicating that FoxO3a is able to restore the apoptotic response to 4-OHT treatment (Figure 3C). Consistently with cell cycle inhibition and apoptosis restoration, proteomics also unveiled the down-regulation of several proteins involved in GF signaling (Figure 3D) such as the Akt family members (AKT1, AKT2 and AKT3) and several PI3K catalytic subunits. In addition, other proteins involved in GF pathways, such as IRS1, SHC1 and RAF1, were markedly reduced. Altogether, proteomic analysis indicates that nuclear FoxO3a restores the sensitivity to 4-OHT by curbing the hyperactive GF signals, notoriously one of the main features of a tamoxifen-resistant phenotype.

### 2.6. FoxO3a Over-Expression Inhibits TamR-Derived Tumor Growth

**Figure 4:**
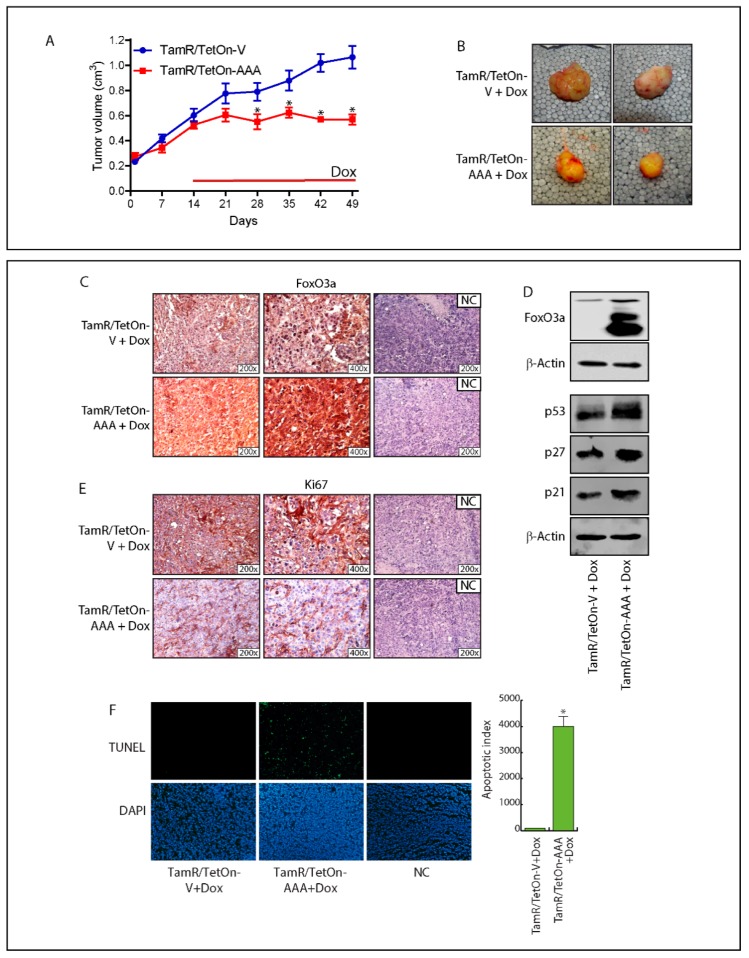
FoxO3a over-expression inhibits the growth of engineered TamR/TetOn derived tumor xenografts. (**A**) TamR/TetOn-V and TamR/TetOn-AAA cells were injected subcutaneously in female nude mice (see Materials and Methods). When the tumors reached average ~0.2 cm^3^, mice were treated with Doxycycline hyclate (Dox) for 35 days. Tumor growth was monitored by caliper, measuring the visible tumor sizes at indicated time points. Data represent the mean ± SD of measurements. * *p* < 0.05 vs. TamR/TetOn-Vector xenografts. (**B**) At the end of the experiment, tumors were explanted and representative tumor images are shown. The expressions of FoxO3a (**C**) and Ki-67 (**E**), as well as the apoptotic index (TUNEL assay) (**F**) were evaluated in tumor sections deriving from Dox-treated mice injected with engineered TamR/TetOn cells. NC: negative control. (**D**) An amount of 50 µg of lysates from explanted tumors were subjected to WB analysis to evaluate Dox-induced FoxO3a overexpression. β-actin was used as the loading control.

The results obtained in vitro have been confirmed in vivo. Mice bearing TamR/TetOn-AAA derived xenografts were administered with Dox to induce FoxO3aAAA expression within the tumor. Over 50% of mass reduction was observed in TamR/TetOn-AAA implants, compared to TamR/TetOn-V derived tumors (Figure 4A,B and Figure S7). The anti-proliferative effect of Dox-induced FoxO3aAAA (Figure 4C,D) was paralleled by the induction of p53, p21 and p27 protein expression (Figure 4D) and a reduced expressions of the proliferation marker Ki-67 (Figure 4E). Finally, TUNEL experiments conducted on tumor sections confirmed that the tumor mass shrinkage was due to FoxO3AAA-induced apoptosis (Figure 4F).

### 2.7. In Silico Validation of the Clinical Relevance of FoxO3a in Human BC Patients

**Figure 5:**
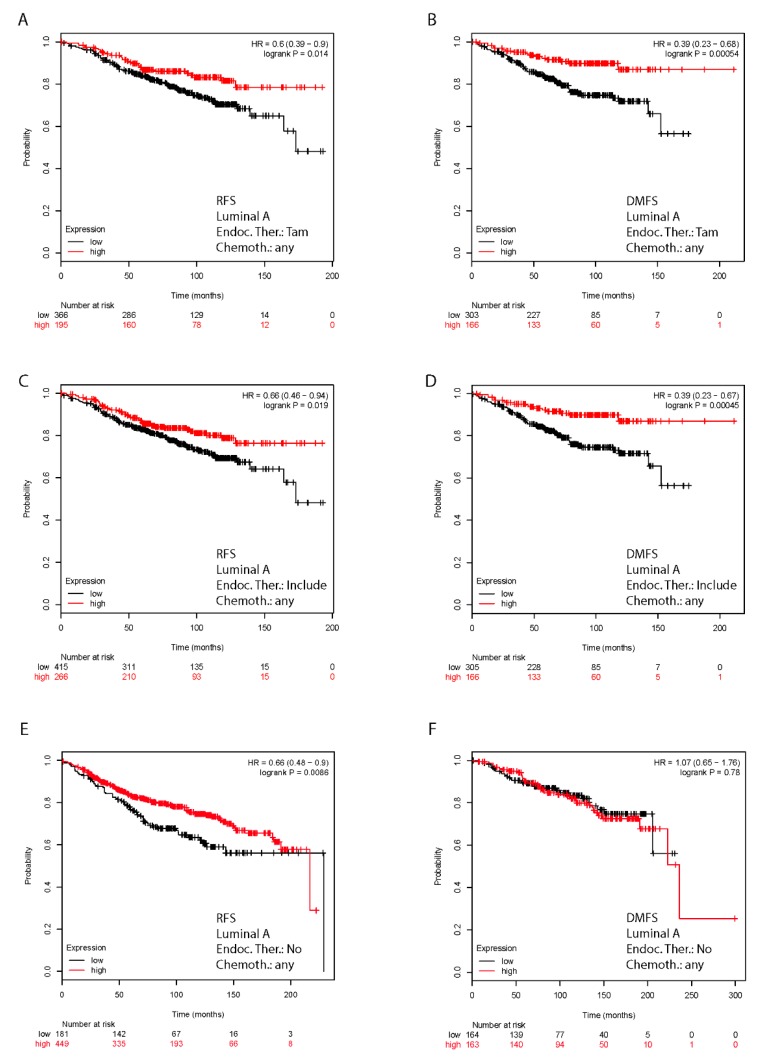
Kaplan–Meier analysis: FoxO3a expression is a positive prognostic factor in Luminal A BC patients. Relapse-free survival (RFS) and distant metastasis free survival (DMFS) were evaluated in a cohort of Luminal A sub-type patients treated with Tam (Endoc. Ther.: Tam) [RFS - 561 patients (**A**), DMFS - 469 patients (**B**)] and treated with unspecified endocrine therapeutics (Endoc. Ther.: Include) [RFS - 681 patients (**C**), DMFS - 471 patients (**D**)]. Luminal A patients not receiving endocrine therapeutics (Endoc. Ther.: No) have been also analyzed [RFS - 630 patients (**E**), DMFS - 327 patients (**F**)]. K–M analysis was performed regardless of a specific chemotherapeutic treatments (Chemoth.: any). K–M are plotted for high (above median, in red) and low (below median, in black) FoxO3a gene expression. Biased and outlier array data were excluded from the analysis. Hazard-ratios were calculated, at the best auto-selected cut-off, and p-values were calculated using the logrank test and plotted in R.

The prognostic value of FoxO3a expression and its clinical relevance in the acquisition of tamoxifen resistance was evaluated *in silico* in a tamoxifen treated human breast cancer cohort of patients, with long-term follow-up. The Kaplan–Meier (K–M) relapse-free survival (RFS) curve (log-rank test, *p =* 0.014) and the distant metastasis free survival (DMFS) curves (log-rank test, *p =* 0.00054) (Figure 5A,B) evidenced a better prognosis for Luminal A subtype breast cancer patients expressing high levels of FoxO3a mRNA. Moreover, the overall survival seems to be improved by FoxO3a expression, but this result is only indicative due to the small number of patients. Similar results were obtained considering a cohort of patients receiving any kind of endocrine therapeutics, not strictly tamoxifen (Figure 5C,D). Thus, low levels of FoxO3a could be used to identify high-risk luminal A. sub-type BC patients that might benefit from potential adjuvant treatments able to increase FoxO3a expression, restoring the sensitivity to tamoxifen therapy. Notably, higher FoxO3a expression lowers the risk of relapse even in patients receiving only chemotherapy (log-rank test, *p =* 0.0086) and not endocrine therapy (Figure 5E). This is not surprising considering that many genotoxic and cytotoxic chemotherapeutic drugs act by inhibiting the PI3K-Akt signaling pathway and that its deregulation, very likely also through FoxO3a inactivation, often contributes to chemotherapy resistance [35]. However, in the same subset of patients, FoxO3a does not seem to lower the risk of distant metastasis (Figure 5F, log-rank test, *p =* 0.78). Similarly, FoxO3a does not show a protective role in Luminal B (Figure S8A,C) and Basal-like (Figure S8B,D) BCs, where, although the results are statistically not relevant, a high FoxO3a expression seems rather to be associated to a lower probability of RFS and DMFS (Figure S8).

### 2.8. The AED Lamotrigine Restores the Sensitivity to Tamoxifen Treatment through FoxO3a Re-Expression in Tamoxifen Resistant Xenografts

Recently published data from our laboratory suggest that a promising pharmacological candidate for such an adjuvant therapy in patients who result refractory to the antiestrogen treatment is LTG, a well-known AED that is able to increase FoxO3a levels in BCCs [28]. Thus, to evaluate if LTG, by inducing FoxO3a expression, is able to restore the sensitivity to the anti-hormonal therapy also in vivo, female nude mice bearing TamR cells-derived tumor xenografts into the intrascapular region, were treated with LTG (20 mg/kg/day) on the basis of our *in vitro* results [28] and on the pertinent literature [36].

**Figure 6:**
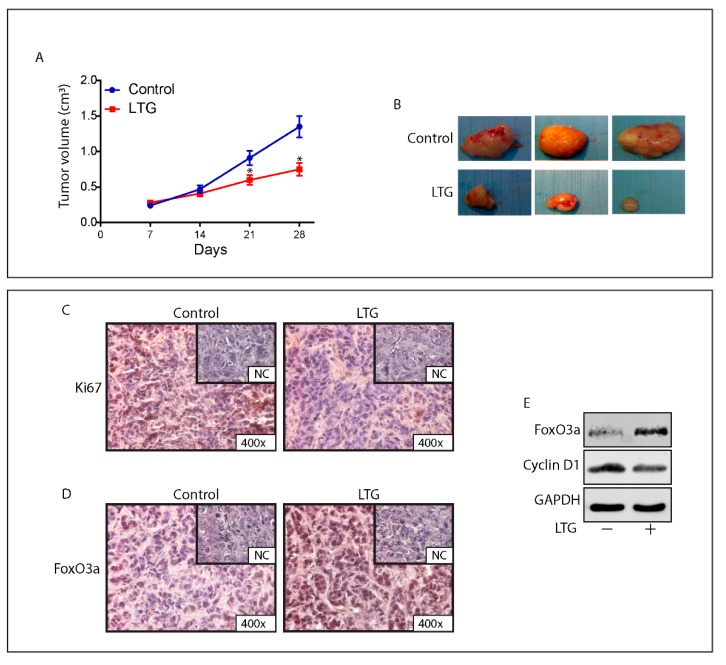
LTG induces FoxO3a and inhibits the growth of TamR derived tumor xenografts. (**A**) Xenografts were established with TamR cells in female mice implanted with E2 and successively, tamoxifen pellets (see Materials and Methods for details). One group was treated with 20 mg/kg/day LTG (*n =* 5) and a second group with vehicle alone (*n =* 5). Tumor mass was measured at indicated time points with a caliper. Data represent the mean ± SD of measurements. * *p* < 0.05 vs. control xenografts. (**B**) Representative images of explanted tumors at day 28. Tumor sections from mice at 28 days were formalin fixed, paraffin embedded, sectioned, and immunostained with hematoxylin and eosin Y (H&E) or incubated with antibodies directed against the epithelial marker cytokeratin 18 (Cyt 18) (Figure S9). Immunostaining was also performed for the proliferation marker Ki-67 (**C**) and for FoxO3a (**D**). (**E**) FoxO3a and CyclinD1 expression was assessed in protein extracts from xenografts excised from control mice and LTG treated mice. GAPDH was used as the loading control.

At sacrifice, no significant difference in the mean weights or histologic features of the major organs (liver, lung, spleen, and kidney) was observed between vehicle-treated mice and those that received LTG treatment, while a significant reduction in tumor volume (~50%) was observed in mice administered with 20 mg/kg/day LTG (Figure 6A,B).

The antiproliferative effect of LTG was confirmed by a reduced expression of the proliferation marker Ki-67 in LTG treated xenografts compared to that observed in tumors deriving from control mice (Figure 6C). These results are congruent with the strong increase in FoxO3a expression observed in both tissue sections and protein extracts from LTG treated tumors respect to control tumors (Figure 6D,E). Moreover, Cyclin D1 downregulation associated to FoxO3a upregulation (Figure 6E) perfectly fits with other authors’ observations [37] and with our proteomics results (Figure 3B). Notably, immunohistochemical analysis of LTG samples clearly shows a markedly nuclear localization of FoxO3a (Figure 6D), which is consistent with an antiproliferative effect. Taken together, these results indicate that LTG might represent a useful tool to be exploited as an adjuvant therapy in patients who failed to respond to tamoxifen treatment.

## 3. Discussion

Approximately 70% of BCs do express ER and are subjected to endocrine-based therapies (selective ER modifiers, e.g., tamoxifen, selective ER down-regulators, e.g., fulvestrant, and aromatase inhibitors, e.g., letrozole, anastrozole and excemestane), but the development of endocrine resistance is highly common and remains an unsolved problem. We addressed our studies on the involvement of FoxO3a in the maintenance of the sensitivity of BC to tamoxifen treatment. Several data from our and others’ laboratories established the existence of a functional interaction between FoxOs and ER in BCCs (reviewed in [16]). In particular, FoxO3a seems to have a protective role in ER+ breast tumors [22,24,38]; therefore, it is reasonable to suppose that FoxO3a deregulation could favor the acquisition of a phenotype resistant to treatments targeting ER, such as tamoxifen.

Here, we report that TamR cells show a strong decrease in FoxO3a expression and in its nuclear localization, if compared to parental, tamoxifen sensitive, cells. Notably, the phenomenon was not peculiar to a specific BCC line (i.e., MCF-7), but it was observed in other tamoxifen resistant BCCs, such as ZR-75-1 and T47D. FoxO3a downregulation in TamR cells was not due to epigenetic modifications on FoxO3a regulatory regions that might stem from a prolonged exposure to Tam [39], but rather to FoxO3a protein degradation. In fact, in TamR cells, FoxO3a is hyper-phosphorylated by hyperactive AKT and MAPK. Hyper-phosphorylated FoxO3a is recognized by the E3 ligase MDM2 [18], which promotes its ubiquitination and degradation. Interestingly, GFs dependent pFoxO3a/MDM2 interaction was evident only in TamR cells exposed to the proteasomal inhibitor MG132, compared to parental cells, confirming that FoxO3a is preferentially targeted for proteasome degradation in TamR. In addition, since FoxO3a can control its own transcription [40], it should not surprise to observe FoxO3a mRNA downregulation in these cells.

Therefore, we questioned whether FoxO3a re-activation might restore the sensitivity to tamoxifen in TamR cells. Indeed, the over-expression of a constitutively active FoxO3a in TamR cells was able to re-establish the anti-proliferative effect of the antiestrogen. Oddly, proteomics and WB analysis did not reveal any significant change in the levels of the pro-apoptotic factors Bim and Fas-L, whose expression is notoriously regulated by FoxO3a [41]. Neither we were ever able to obtain the induction of these two proteins in cells transiently expressing FoxO3aAAA, nor their downregulation in parental cells silenced for FoxO3a. However, other pro-apoptotic proteins like BAD, PARP and several caspases were upregulated by active FoxO3a in TamR cells. Noteworthy, although FoxO3a seems to be involved in the regulation of autophagy [42], proteomic analysis, nor our TEM observations, did not reveal any significant alteration in the expression of mediators of the autophagy response in FoxO3aAAA expressing TamR cells. Interestingly, proteomics also unveiled how FoxO3a affects the expression of additional cell cycle modulators (besides the already known ones [33]), such as CDC16, CDC27, CDK2, CDK4, CDK6 and SFN. The ability of nuclear FoxO3a to restore the sensitivity of breast cancer cell to tamoxifen is finally highlighted by the down regulation of several proteins involved in GF signals, e.g., components of the PI3K/AKT pathway, the major inhibitor of FoxO3a activity, two adaptor proteins IRS1 and SHC1 and the transduction factor RAF1. While IRS members have already been reported to be transcriptionally regulated by FoxOs [43,44], several other proteins modulated by FoxO3a over-expression did not, paving the way for further investigations.

On the contrary, FoxO3a knockdown rescued MCF-7 cells from the antiproliferative effect of tamoxifen, mimicking the behavior of TamR cells, which express low FoxO3a levels. The mechanism through which FoxO3a mediates the cellular response to the antiestrogen in sensitive BCCs is currently under investigation in our laboratory, although, in our cell model, we failed to observe the involvement of the G protein-coupled estrogen receptor (GPER), as recently described [45].

The K–M analysis conducted on a Luminal A subtype population of breast cancer patients subjected to tamoxifen therapy corroborated our observations, showing that high levels of FoxO3a strongly correlate with a positive response to tamoxifen treatment and consequently, with a long-term relapse free survival. Thus, FoxO3a expression might not only be considered a favorable prognostic marker in breast cancer, as described elsewhere [24,46] but, even more importantly, it might also be predictive of the potential efficacy of the antiestrogen therapy. Nevertheless, it cannot be excluded that the low levels of FoxO3a observed in ER+ BC patients might be, at least for the subgroup receiving tamoxifen therapy, a consequence of the gradual acquisition of a tamoxifen resistant phenotype. On the other hand, RFS and DMFS curves are not significantly affected by FoxO3a expression in Luminal B or Basal-like BC cohorts of patients. Therefore, FoxO3a seems to be a positive prognostic factor for Luminal A BC, but not for Luminal B or Basal-like subgroups. In this regard, it is worth underlining that the cell lines used in this work are all classified as Luminal A [47]. This assessment is in line with our and others’ previous findings, where FoxO3a shows a protective role in Luminal-like [22,24], but not in triple negative breast tumors [22,48]. Therefore, a more accurate study on FoxO3a protein expression by means of IHC of tissue microarrays (TMA), or even proteomics, in all the different BC subtypes is mandatory, in order to rule out the significance of FoxO3a as a predictive marker when associated (or not) with other prognostic factors.

Several conventional drugs and chemical entities, mainly acting as inhibitors of the tyrosine kinase pathways, have been suggested to increase FoxO3a nuclear accumulation, thus promoting apoptosis in several cancer cell lines [21]. Moreover, four Phase 1–2 clinical trials on BC patients, using PI3k/AKT inhibitors in combination or not with the pure antiestrogen fulvestrant (ClinicalTrials.gov Identifier: NCT01339442, NCT02260661 and NCT02077569) or a combination of the anticancer drug paclitaxel and the IL-8 receptor antagonist reparaxin (Identifier: NCT02001974) aimed to assess, as a secondary endpoint, FoxO3a expression, just ended. Unfortunately, the results of these interventional studies are still not available. Further, many kinase inhibitors are associated with toxicities and off-target effects such as cardiotoxicity, hypertension, hypothyroidism, hematological disorders and others [49].

Here we add a new molecule able to restore the anti-proliferative response to tamoxifen treatment of ER+ BCCs, LTG (lamotrigine, Lamictal) [50], a well-known AED. The use of AEDs as potential anti-cancer agents against several types of cancer, including breast, has been widely reported [26,27]. In particular, we recently demonstrated that LTG, by inhibiting the PI3K/Akt signaling, increases the expression and nuclear localization of FoxO3a [28].

In agreement with these findings, a recent prospective comparative study aimed at evaluating the outcome of cancer patients treated or not with AEDs to prevent seizures, identified a better overall survival for patients receiving AEDs, including LTG [51].

**Figure 7:**
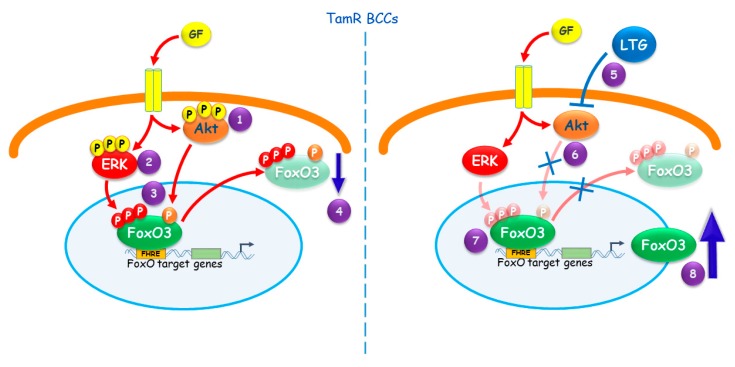
Proposed model for FoxO3a role in tamoxifen resistant BCCs. The hyper-activated AKT (1) and ERK (2) pathways in TamR cells cause (3) FoxO3a sustained phosphorylation, leading to (4) its proteasomal degradation. (5) The AED LTG restores the response to tamoxifen in TamR BC by inducing FoxO3a. This occurs since LTG, inhibiting the PI3K/Akt pathway (6), re-activates FoxO3a (7), increasing its own transcription (8) [28].

Although repositioning of old drugs is not economically convenient for Pharma industries, we strongly encourage a great effort in that direction, since starting from existing and clinically approved molecules can significantly reduce the cost and the time required for the development of new anticancer molecules, making these therapies rapidly available to patients who are in urgent need of cure. Noteworthy, LTG showed only a mild toxicity, comparable to control patients, in a phase 3 clinical trial, aimed at evaluating effect on chemotherapy-induced peripheral neuropathy (CIPN) [52]. However, tamoxifen co-administration has been reported to increase the serum levels of some AEDs, especially phenytoin, most likely by inhibiting their metabolism [53], although, as far as we know, interference with LTG metabolism has been only described *in vitro* and not in patients [54]. Therefore, an accurate pharmacokinetic evaluation is mandatory in order to avoid the risk for LTG under-treatment or else toxicity (mainly skin rash and changes in vision). Conclusions are summarized in Figure 7. Our results emphasize the need for developing anti-cancer therapies exploiting FoxO3a in BC also in those patients with acquired resistance to tamoxifen treatment. Indeed, FoxO3a reactivation seems to be a promising tool to restore the sensitivity to the antiestrogen in Luminal A BC patients.

## 4. Materials and Methods

### 4.1. Cell Cultures, Conditions, and Treatments

ER+ human breast cancer epithelial cell line MCF-7 was purchased from ATCC (LGC Standards S.r.l., Milan, Italy) and used within 4 months after frozen aliquots resuscitations (less than 30 passages). Every 4 months, cells were authenticated by short tandem repeat analysis (AmpFLSTR Profiler Plus PCR Amplification Kit, Applied Biosystems Monza Brianza, Italy) at our Sequencing Core. Morphology, doubling times, estrogen sensitivity, and mycoplasma negativity (MycoAlert, Lonza, ThermoFisher Scientific, Milan, Italy) were tested. Cells were cultured in DMEM/F-12, supplemented with 5% fetal bovine serum (FBS), 100 IU/mL penicillin, 100 ng/mL streptomycin, and 0.2 mM l-glutamine. For experimental purposes, cells were synchronized in phenol red-free and serum-free media (PRF-SFM) for 24 h and then, where opportune, switched to PRF-media containing 5% charcoal-treated FBS (PRF-CT) or 5% FBS, in the presence or not of tamoxifen (4-OHT), EGF or MG132 (all from Sigma-Aldrich, Merck, Milan, Italy) depending on the experiment. All other media and reagents were purchased from ThermoFisher Scientific, Milan, Italy.

### 4.2. RNA Extraction, Reverse Transcription, and Real-Time (RT)-PCR

Total RNA was isolated using TRI-reagent (ThermoFisher Scientific), treated with DNase I (Life Technologies, ThermoFisher Scientific) and reverse transcribed (2 μg) with the High-Capacity cDNA Reverse Transcription Kit (ThermoFisher Scientific) according to the manufacturer’s instructions. cDNA was diluted 1:3 in nuclease-free water and 5 μL was analyzed in triplicate by RT-PCR in a iCycler iQ (Bio-Rad, Milan, Italy) using SYBR green Universal PCR Master Mix (Bio-Rad) and the following pairs of primers: FOXO3 forward 5′-CAAACCCAGGGCGCTCTT-3′ and reverse 5′-CTCACTCAAGCCCATGTTGCT-3′. Negative controls contained water instead of cDNA. Each sample was normalized on its 18S rRNA content (ThermoFisher Scientific). Relative gene expression levels were normalized to a calibrator that was chosen to be the basal, untreated sample. The final results were expressed as n-fold differences in gene expression relative to 18S rRNA and the calibrator, calculated as described previously [22].

### 4.3. Western Blotting (WB) Assay

Total, cytosolic and nuclear proteins were extracted and counted as previously described [23]. Lysates were separated on SDS-PAGE gel transferred to nitrocellulose membranes and proteins were detected with specific polyclonal (p) or monoclonal (m) antibodies (Abs), recognized by IRDye secondary Abs (LI-COR Corporate, Milan, Italy). The Abs employed were anti-FoxO3a (75D8, #2497), p-FoxO3a (Ser253; #13129), p-FoxO3a (Ser294; #5538), p44/42 MAPK (#9102), Phospho-p44/42 MAPK (Thr202/Tyr204) (#9101) (all from Cell Signaling Technology Europe, B.V., Leiden, The Netherlands). MDM2 (IF2; #33-7100) (ThermoFisher Scientific), AKT 1/2/3 (H136; sc-8312), p-AKT 1/2/3 (Ser473) (sc-7985), p21 (sc-71811), p27 (sc-53871), p53 (sc-126), CD1 (sc-718), β-Actin (AC-15; sc-69879), GAPDH (FL-335; sc-25778) and Lamin B (C-20; sc-6216) all from Santa Cruz Biotechnology, Inc., Heidelberg, Germany. Images were acquired with the Odyssey FC Imaging System (LI-COR Corporate). For each Western blot figure, the whole blots showing all the bands with all molecular weight markers, as well as the densitometry readings/intensity ratio of each band (analyzed using Image J software, NIH, USA) are collected in the Supplemental Materials as a separate file named “Original blots and densitometry”.

### 4.4. Immunostaining

MCF-7 and the derived tamoxifen resistant (TamR) cells were seeded in growing medium on coverslips. The next day, cells were fixed with 3% paraformaldehyde and permeabilized with 0.2% Triton X-100. Non-specific sites were blocked with bovine serum albumin (BSA) (3% for 30 min). Samples were incubated for 1 h with anti-FoxO3a (75D8) Ab (2 µg/mL), washed and incubated with fluorescein-conjugated goat anti-rabbit IgG (Sigma-Aldrich, Merck) secondary Ab. 4′,6-Diamidino-2-phenylindole (DAPI, Sigma-Aldrich, Merck) was used to counterstain nuclei. Captures were taken at ×400 magnification using ViewFinder™ 7.4.3 Software connected to an Olympus camera p50. Optical densities of stained FoxO3a proteins were analyzed by ImageJ software.

### 4.5. Proximity Ligation Assay (PLA)

PLA was performed using a Duolink In Situ Detection Kit (Sigma-Aldrich, Merck) as recommended by the manufacturer. PLA probes were chosen to detect mouse (MDM2) and rabbit (FoxO3a) Abs. Samples were mounted on slides using a DUOLINK In Situ Mounting Medium with DAPI (for nuclear staining) and observed under a laser scanning confocal microscope (Olympus FV3000; Olympus Italia, Milan, Italy).

### 4.6. Generation of FoxO3a Inducible Stable Cell Lines

TamR/TetOn-AAA cells and the corresponding control cell (TamR/TetOn-V) were generated using the Tet-On Gene Expression System (Clontech, Ancona, Italy). A detailed description of the cloning process can be found in the Supplementary Information.

### 4.7. siRNA-Mediated RNA Interference

Validated Stealth RNAi™ siRNA-annealed duplexes (Oligo ID: VHS41092) were used for effective depletion of FoxO3a (siFoxO3a) transcripts. A Stealth RNAi™ siRNA (siScramble) lacking identity with known gene targets was used as a negative control. MCF-7 cells were grown in medium without antibiotics for 24 h and transfected with siFoxO3a (150 pmol/dish) and siScramble (120 pmol/dish), using Lipofectamine 2000 (Invitrogen, ThermoFisher Scientific). Six hours later, cells were synchronized in PRF-SFM for 24 h and switched to 5% PRF-CT in the presence or absence of 1 µM 4-OHT for up to 72 h, depending on the experiment.

### 4.8. Growth Assay

Cells were plated in 12-well plates (10^5^ cells/well) in GM-PRF. After 16 h, cells were synchronize in PRF-SFM for 24 h (day zero) and then shifted in PRF-CT and treated or not with Doxycycline (Dox) (1 µg/mL) for 24, 48 and 72 hours. Cells were harvested by trypsinization, incubated in a 0.5% trypan blue solution and counted through a Countess^®^ II Automated Cell Counter (Life Technologies, ThermoFisher Scientific). 4-OHT (1 µM) treatment was refreshed every day.

### 4.9. Cell Cycle Analysis

Cells were seeded at 1 × 10^6^ cells in 60 mm plates and treated as described for growth experiments for 72 h. Cells were harvested, permeabilized with 75% ethanol, treated with 1 mg/mL RNase A, stained with 50 μg/mL propidium iodide and analyzed by flow cytometry (FACScan, Becton Dickinson, Milan, Italy). Data were analyzed as previously described [55].

### 4.10. Transmission Electron Microscopy (TEM)

TEM was conducted as previously described [56]. Cells were seeded in 60 mm Petri dishes and then treated as described for growth experiments for 72 h. Cells were fixed in 3% glutaraldehyde (Sigma-Aldrich, Merck) solution in 0.1 M phosphate buffer (pH 7.4). Then the samples were post-fixed in osmium tetroxide (3%), dehydrated in graded acetone, and embedded in Araldite (Sigma-Aldrich, Merck). Ultrathin sections were collected on copper grids and contrasted using both lead citrate and uranyl acetate. Grids were examined in a Zeiss EM 10 electron microscope (ZEISS, Oberkochen, Germany).

### 4.11. TUNEL Assay

Cells (3 × 10^5^) were seeded on coverslips in 35 mm Petri dishes and treated as described for growth experiments. After 72 h, apoptosis was determined by enzymatic labelling of DNA strand breaks using a Dead End Fluorimetric TUNEL System (Promega, Milan, Italy) according to the manufacturer′s instructions. Coverslips were mounted on slides using Fluoromount mounting medium (Sigma-Aldrich, Merck) and observed under a fluorescence microscope (Olympus BX51). DAPI was used to counterstain the nuclei. Apoptotic cells were photographed at 10x magnification using an Olympus dp50 camera and ViewFinder software and counted using Image J software.

### 4.12. Label-Free Semi-Quantitative Proteomic Analysis

Cell lysates were prepared for trypsin digestion by sequential reduction of disulphide bonds with TCEP and alkylation with MMTS. Then, the peptides were extracted and prepared for LC-MS/MS. All the LC-MS/MS analyses were performed on an LTQ Orbitrap XL mass spectrometer (ThermoFisher Scientific) coupled to an Ultimate 3000 RSLCnano system (ThermoFisher Scientific). Xcalibur raw data files acquired on the LTQ-Orbitrap XL were directly imported into Progenesis LCMS software (Waters Corp, Milan, Italy) for peak detection and alignment. Data were analyzed using the Mascot search engine. Five technical replicates were analyzed for each sample type [57].

#### Ingenuity Pathway Analysis (IPA)

Pathway and function analyses were generated using Ingenuity® Pathway Analysis (IPA®, Qiagen, Hilden, Germany) (Ingenuity systems, http://www.ingenuity.com), which assists with proteomics data interpretation via grouping differentially expressed genes or proteins into known functions and pathways. Pathways with a z score > 2 were considered as significantly activated and pathways with a z score < −2 were considered as significantly inhibited.

### 4.13. Kaplan–Meier Analysis

The prognostic value of FoxO3a in breast cancer was evaluated by performing a Kaplan–Meier (K–M) analysis using the most updated version of a publically available microarray database from breast cancer patients (http://kmplot.com/analysis/index.php?p=service&cancer=breast). Relapse-free survival (RFS) and distant metastasis free survival (DMFS) were evaluated in a cohort of Luminal A sub-type patients treated with Tam only (RFS = 561 and DMFS = 469 patients) and in a cohort of Luminal A sub-type patients treated with any endocrine therapeutics, i.e., tamoxifen, aromatase inhibitors and ER disruptors, such as fulvestrant (RFS = 681 and DMFS = 471 patients), regardless of chemotherapeutic treatments. A cohort of patients untreated with endocrine therapeutics (RFS = 630 and DMFS = 327 patients) was used as control. RFS and DMFS were also evaluated in a cohort of Luminal B and Basal-like sub-type patients. The search parameters used are reported in the *Supplementary Materials and Methods*. The *p*-values were calculated using the log rank test and plotted in R as reported by Gyorffy B. et al. [58].

### 4.14. In Vivo Studies TamR/TetOn Derived Xenografts

Female 45 day-old athymic nude mice (nu/nu Swiss; Harlan Laboratories, MB, Italy) were maintained in a sterile environment. At day 0, estradiol pellets (0.72 mg/pellet, 90-day release; Innovative Research of America, Sarasota, FL, USA) were subcutaneously implanted into the intrascapular region of the mice. The next day, exponentially growing TamR/TetOn-V or TamR/TetOn-AAA cells (5.0 × 10^6^ per mouse) were inoculated subcutaneously in 0.1 mL Matrigel (BD Biosciences, Milan, Italy). When the tumors reached average ~0.2 cm^3^ (i.e., in about 4 weeks), mice were allocated to estrogen withdrawal plus tamoxifen (n = 5 mice per group; estradiol pellets were removed and tamoxifen pellets (5 mg/pellet, 90-day release) were implanted to each mouse into the intrascapular region). After two weeks, the mice were divided into two groups, according to engineered TamR/TetOn cells injection.

Doxycycline hyclate (Sigma Aldrich, Merck) 2 g/L was delivered to the mice through drinking water (tap water + 3.0% sucrose (Sigma Aldrich, Merck) in dark stained bottles and renewed every 3 days for 35 days.

TamR/TetOn derived xenograft tumor growth was monitored twice a week by caliper measurements as described [28]. At day 49, animals were sacrificed following the standard protocols and tumors were dissected from the neighboring connective tissue. A part of the tumors was frozen in nitrogen and stored at −80 °C; the remainder of each sample was either fixed in 4% formalin and embedded in paraffin for the histologic analyses or lysed.

### 4.15. In Vivo Studies TamR Derived Xenografts

Female 45-day-old athymic nude mice were processed as described above (Section 4.14). TamR cells were inoculated subcutaneously. Two weeks after the removal of estradiol pellets and the implant of tamoxifen pellets, mice were divided into two groups: the first (*n* = 5) receiving vehicle and the second (*n* = 5) LTG (20 mg/kg/day). Mice were treated, monitored and sacrificed as previously described [28].

#### Animal Care

Mice well tolerated all *in vivo* procedures, since no changes in body weight, motor function or food and water consumption were observed. Animal care, death, and experiments were done in accordance to the principle of the 3Rs and according to Italian law (D.L. 26/2014), the Guide for Care and Use of Laboratory Animals published by the US National Institutes of Health (2011), and the Directive 2010/63/EU of the European Parliament on the protection of animals used for Scientific research. The animal research project was approved by the Italian Ministry of Health, Rome (authorization n. 559/2015-PR), ethical code 26437.EXT.3. All the experimental procedures were approved by the Bioethics Committee on Animal Experiments (OPBA) of the Department of Pharmacy, Health and Nutritional Sciences, University of Calabria, n° 62 27/11/2014 and n° 85 10/12/2015.

### 4.16. Histological Analysis

Formalin-fixed paraffin-embedded sections of tumor xenografts were stained as previously reported [28]. The epithelial nature of the tumors was verified as previously described [28].

### 4.17. Statistical Analysis

All data were expressed as the mean ± standard deviations (SD) of at least three independent experiments. Statistical significances were evaluated using Student’s *t* test.

## 5. Conclusions

Our results, summarized in Figure 7, emphasize the need for developing anti-cancer therapies exploiting FoxO3a in BC, also in those patients with acquired resistance to tamoxifen treatment. Indeed, FoxO3a reactivation seems to be a promising tool to restore the sensitivity to the antiestrogen in Luminal A BC patients.

## Supplementary Materials

The following are available online at https://www.mdpi.com/2072-6694/11/12/1858/s1, Figure S1: Growth curve response to tamoxifen of TamR BCCs with respect to sensitive parental cells; Figure S2. FoxO3a resensitizes Tam resistant ZR-75-1 e T47D BCCs to tamoxifen treatment; Figure S3. FoxO3a interacts with MDM2 in TamR BCCs; Figure S4. MG132 treatment inhibits FoxO3a and MDM2 protein degradation; Figure S5. FoxO3a decreases the proliferation of TamR breast cancer cells; Figure S6. TEM observations on Dox-treated TamR/TetOn-AAA supernatants; Figure S7. Histologic analysis of TamR/TetOn derived tumor xenografts; Figure S8. Kaplan–Meier analysis: FoxO3a loses its positive prognostic value in Luminal B and Basal-like BC patients; Figure S9. Histologic analysis of TamR derived tumor xenografts treated or not with LTG.
