# Supplementary material for: FoxO3a as a Positive Prognostic Marker and a Therapeutic Target in Tamoxifen-Resistant Breast Cancer
Source: Cancers (Basel). 2019 Nov 25;11(12):1858. doi: 10.3390/cancers11121858 (PMC6966564; doi:10.3390/cancers11121858)
Supplement: Supplementary file 1 [file cancers-11-01858-s001.zip › cancers-649799-suppl-layout (DS).docx]

Supplementary Materials: FoxO3a as a Positive Prognostic Marker and a Therapeutic Target in Tamoxifen-Resistant Breast Cancer

Michele Pellegrino, Pietro Rizza, Ada Donà, Alessandra Nigro, Elena Ricci, Marco Fiorillo, Ida Perrotta, Marilena Lanzino, Cinzia Giordano, Daniela Bonofiglio, Rosalinda Bruno, Federica Sotgia, Michael P. Lisanti, Diego Sisci and Catia Morelli

Methods

Cell Cultures

The ER+ human BC epithelial cell line ZR-75-1 were maintained in monolayer culture in Dulbecco’s modified Eagle’s/Ham’s F-12 medium (1:1) (DMEM/F-12), supplemented with 5% fetal bovine serum (FBS). T47D cells were cultured in RPMI containing 10% FBS, 2.5 g/ml glucose, 1% Na-Pyruvate, 10 nM Hepes, and 0.2 U/ml insulin. Additionally, culture media were supplemented with 100 IU/ml penicillin, 100 ng/ml streptomycin, and 0.2 mM L-glutamine.

Cells have been purchased from ATCC (LGC Standards, Milan Italy). All media and reagents were purchased from Invitrogen (ThermoFisher Scientific, Milan, Italy).

Generation of Tamoxifen Resistant Cell Lines

MCF-7/TR (TamR), ZR-75-1 /TR and T47D/TR cell lines were selected after long-term cultivation of parental ERα+ cells (MCF-7, ZR-75-1 and T47D, respectively). To induce resistance to tamoxifen, cells were exposed stepwise against increasing concentrations of 4-hydroxytamoxifen (4-OHT, by Sigma-Aldrich, Merck, Milan, Italy), starting from 10^-9^M up to a final concentration of 10^-6^M. Cells were refeed with fresh growth medium containing the drug every 2-3 days. The acquired resistance to the antiestrogen was checked on a scheduled basis by the lack of any inhibitory effect on the proliferation of resistant cell lines compared to the parental ones (**Fig. S1**).

Generation of FoxO3a Inducible Cell Lines

TamR/TetOn-AAA cells were generated using the Tet-On Gene Expression System (Clontech, Palo Alto, CA, USA) that employs a regulator plasmid and a response plasmid to establish a double-stable Tet cell line. The Tet-On system allows activation of gene expression by the addition of the effector substance tetracycline or one of its derivatives, e.g. Doxycycline (Dox by Sigma-Aldrich, Merck, Milan, Italy).

To generate pTRE-F3aAAA inducible plasmid, the cDNA encoding the entire open reading frame of a constitutively active form of the human FoxO3a gene, where the three known AKT phosphorylation sites on FoxO3a have been mutated to alanine (F3aAAA), was excised as a 2kb BamHI-XbaI fragment from the plasmid 1319 pcDNA3 flag FKHRL1 AAA (Addgene, Cambridge, MA, USA, plasmid #10709). The fragment was sub-cloned into the pTRE-zeo vector, harbouring a EGFP cassette and a Zeocin resistance gene to allow selection of transformed cells in the presence of zeocin (Gibco, ThermoFisher, Milan, Italy). Restriction digestion and sequence analysis verified a correct cloning. To obtain a TamR/TetOn-AAA cell line, TamR cells were first transfected with the regulator plasmid pTet-On, containing the Geneticin (G418, Invitrogen, Milan, Italy) resistance gene, using FuGENE HD Transfection Reagent (Promega Italia, Milan, Italy).

Several G418 resistant TamR/TetOn cells were isolated and selected by successful transient transfections with the pTRE-F3aAAA plasmid.

TamR/TetOn selected cells were pooled together and subjected to a second round of stable transfection with the pTRE-F3aAAA plasmid. G418- and Zeocin-resistant cells were isolated and screened by western blot (WB). TamR/TetOn-AAA cells with low background expression and high Dox-dependent (0.5-3 μg/ml) induction of FoxO3a protein were selected. Control cell lines (TamR/TetOn-V) were established by stably transfecting the pTRE backbone (vector only) without a cDNA insert.

Pools of TamR/TetOn-AAA and TamR/TetOn-V cells were collected and used in all experiments.

Cells were maintained in monolayer culture in Dulbecco’s modified Eagle’s/Ham’s F-12 medium (1:1) (DMEM/F-12), supplemented with 5% (FBS), 100 IU/mL penicillin, 100 ng/ml streptomycin, 0.2 mM L-glutamine, G418 (0.2 mg/mL) and Zeocin (0.1 mg/mL).

Plasmids, Transient Transfections and Proliferation Assay

To perform growth curves, ZR-75-1 /TR and T47D/TR were transfected in suspension in GM-PRF with 0,7ug/well of 1319 pcDNA3 flag FKHRL1AAA (F3aAAA), encoding the constitutively active triple mutant of FoxO3a (provided by William Sellers, Addgene plasmid 10709) or the pcDNA3.1 vector (Invitrogen) as control and then plated (10^5^ cells/well) in triplicates 12-multiwell. Transfections were carried out using FuGENE® HD (DNA/ FuGENE ratio, 2:1). After 6 h, the medium was replaced with fresh PRF-SFM, shifted next day to PRF-CT and treated or not for 1, 2 and 3 days with 1µM 4-OHT. Cell viability was determined as described in the main text (“Proliferation assay” in *Materials and Methods*).

Cytosol-Mitochondria Fractionation

For cytosolic and mitochondrial fractionation, cells were lysed (10 min, 4 °C) in cytosolic buffer containing 250 mM Sucrose, 10 mM HEPES (pH 8), 10 mM KCl, 1.5 mM MgCl2, 1 mM EDTA, 1 mM EGTA, 0.1% digitonine and PMSF 1mM (all from Sigma-Aldrich, Merck, Milan, Italy). After centrifugation (13000 rpm, 10 min, 4 °C), supernatants (cytosolic fraction) were collected in separate tubes and the pellets were washed in PBS, centrifuged (13000 rpm, 5 min, 4°C) and incubated for 10 min at 4°C in 10 mM Tris-HCl (pH 7.4), 1 mM EDTA, 1 mM EGTA, 1% Triton X-100 plus inhibitors (all from Sigma-Aldrich, Merck, Milan, Italy). After centrifugation (13000 rpm, 10 min, 4 °C), supernatants containing the soluble fraction of the mitochondrial proteins were collected in separate tubes.

Kaplan-Meier Analysis

K-M analysis was performed as described in *Materials and Methods*. Search parameters used are: Gene: FoxO3a (Affy ID: 204131_s_at and 217399_s_at); Split patients by: auto-select best cutoff; Censure at threshold, selected; Use only JetSet best probe set, selected; Quality control, remove redundant samples: checked; Array quality control: exclude biased arrays selected; Restriction: ER status positive; Intrinsic subtype, selected as indicated.

Statistical analysis

All data were expressed as the mean ± standard deviations (SD) of at least three independent experiments. Statistical significances were evaluated using Student’s t test.


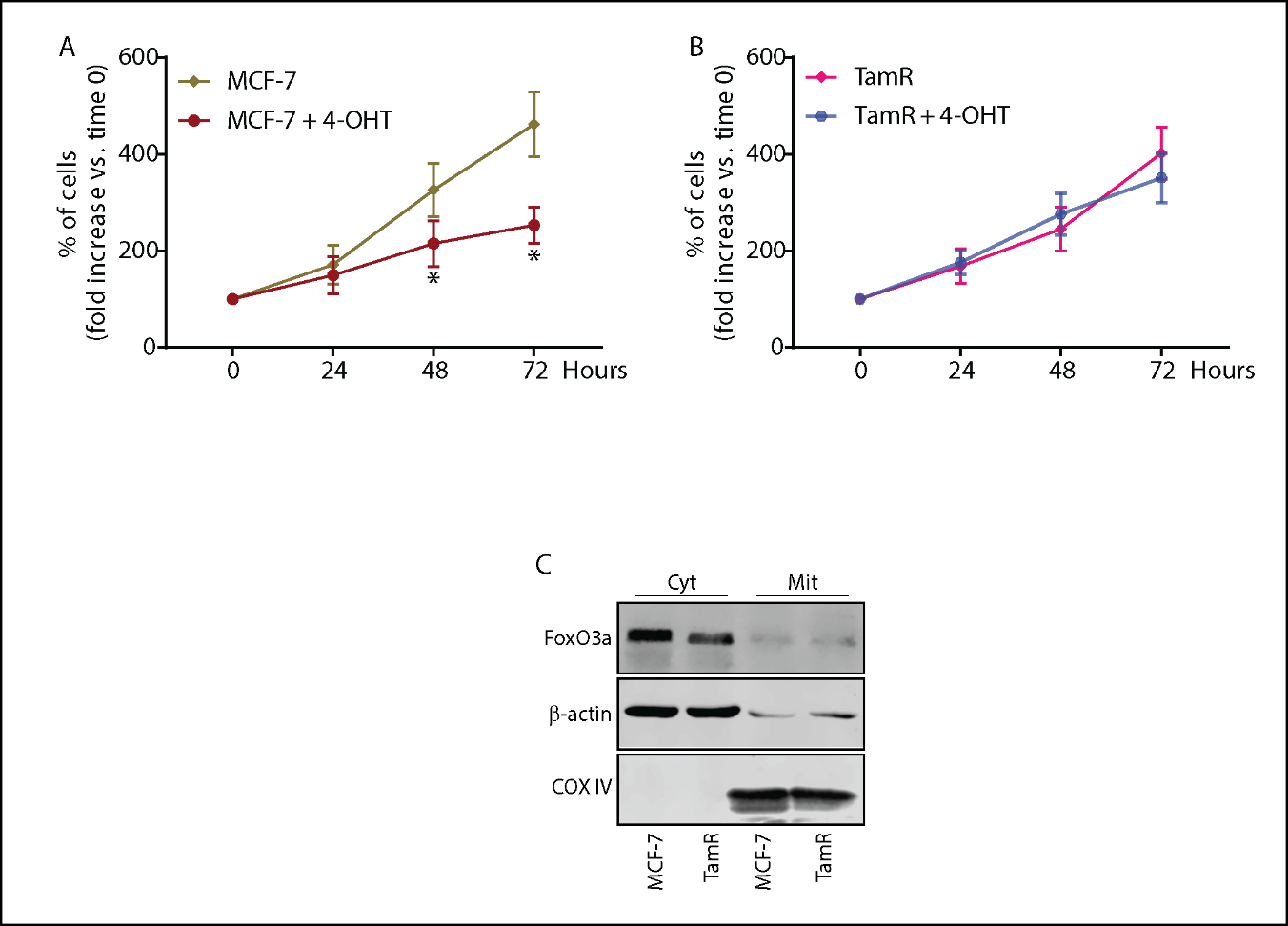


**Figure S1.** Growth curve response to tamoxifen of TamR BCCs with respect to sensitive parental cells. MCF-7 (**A**) and TamR (**B**) cells were starved ON and then switched to 5% PRF-CT and treated or not with 4-OHT 1mM for 24, 48 and 72 h. At each time point, cells were harvested by trypsinization and incubated in a 0.5% trypan blue solution, for 10 min at room temperature. Trypan blue negative cells were counted through a Countess^®^ II Automated Cell Counter (Life Technologies, Italy). 4-OHT (1 µM) treatment was refreshed every day to maintain constant levels in the medium. Data are reported as percentage of cell increase over time 0. Results are the mean ± s.d. of at least three independent experiments. *, *p* < 0.05 vs. untreated. (**C**) Cytosolic and mitochondrial fractions were extracted from growing MCF-7 and TamR cells and subjected to WB analysis to assess the expression of the indicated proteins. β-Actin was used as loading control; COX IV: mitochondrial marker to assess fractionation quality.

**
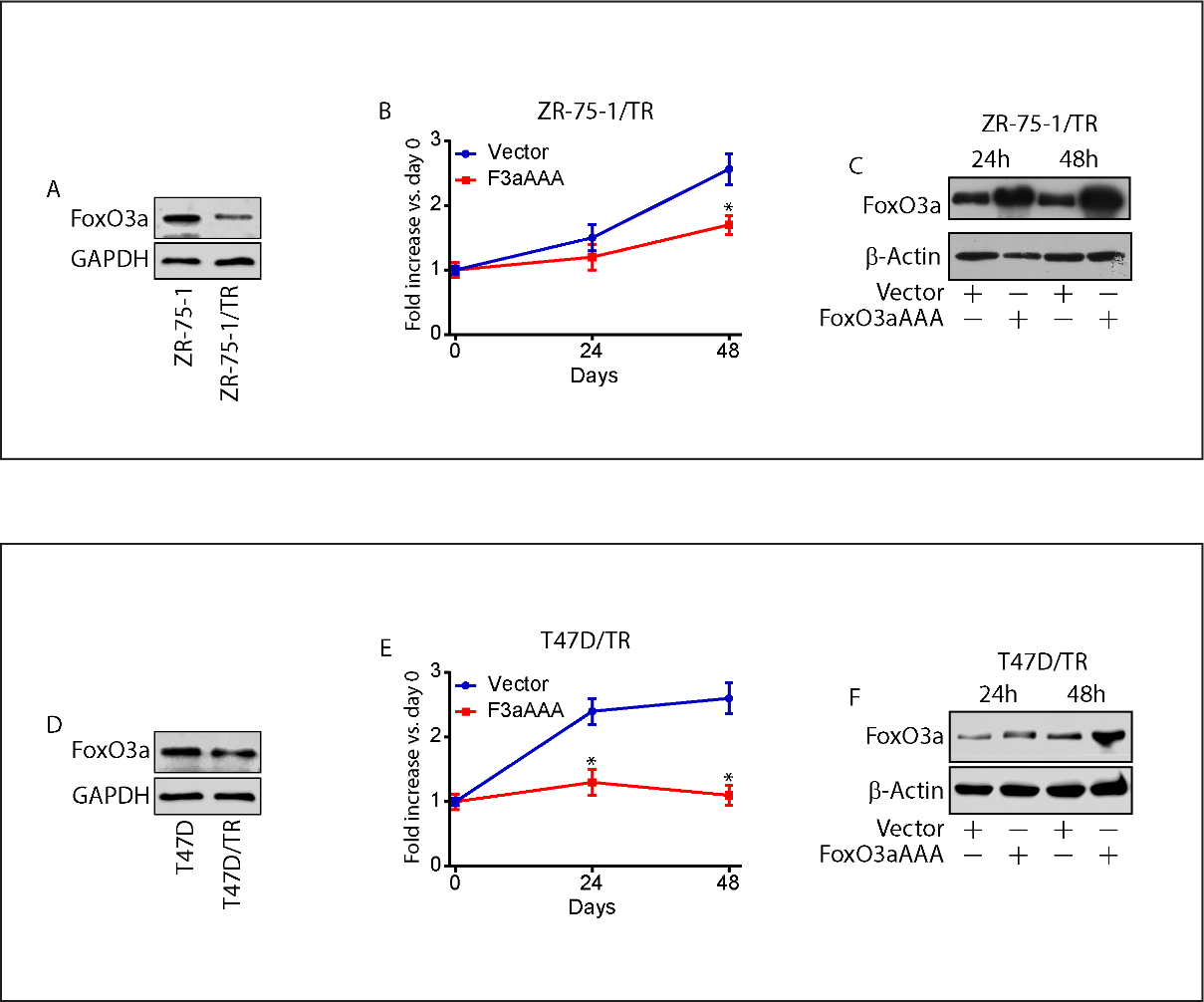
**

**Figure S2.** FoxO3a resensitizes Tam resistant ZR-75-1 e T47D BCCs to tamoxifen treatment. FoxO3a expression was evaluated in ZR-75-1/TR (**A**) e T47D/TR (**D**) cells and in the wild type (WT) parental cells. ZR-75-1/TR (**B**) and T47D/TR (**E**) cells were transfected, as described in *Materials and Methods*, with F3aAAA or pcDNA3.1. Cells were serum starved for 24 h and then switched to 5% PRF-CT plus 1 μM Tam. Tam treatment was renewed every day. Cells were then harvested by trypsin and counted using trypan blue dye exclusion assay after 24 and 48 h (**B**,**E**). Data represent the mean ± SD of three independent experiments. The error bars indicate SD. Duplicate experiments were subjected to WB analysis to assess FoxO3a expression in transfected ZR-75-1 /TR and T47D/TR cells (**C,F**). β-Actin was used as a loading control.

**
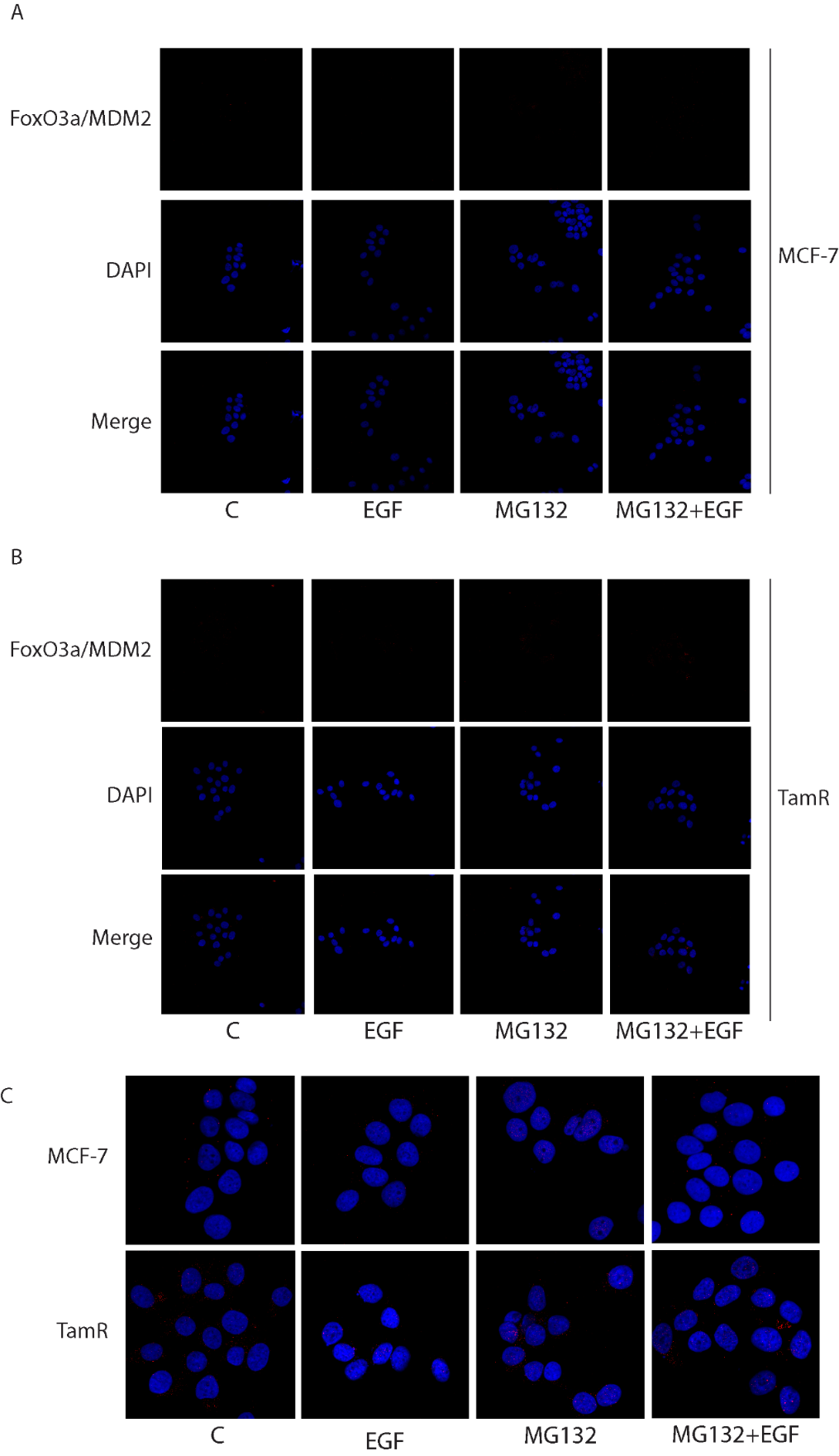
**

**Figure S3.** FoxO3a interacts with MDM2 in TamR BCCs. Duolink was used to detect FoxO3a/MDM2 interaction in MCF-7 (**A**) and TamR (**B**) cells. Cells were seeded in MW8 (Lab-Tek™ Chamber Slide System, Nunc™) and let adhere for 48h. Cells were then starved in PRF-SFM and pre-treated with 20 µM MG132 or left untreated (control, **C**). The next day EGF (100nM) was added for 30 min were indicated. Antibodies against FoxO3a and MDM2 were used to detect the active complexes (Red staining: PLA signal). Blue: DAPI (nuclei). Representative images were taken at ×400 magnification. (**C**) Merged images at a higher magnification. Stained FoxO3a/MDM2 complexes were counted by ImageJ software and their relative abundance *vs* untreated samples is reported in the graph in Fig.1F (main text).


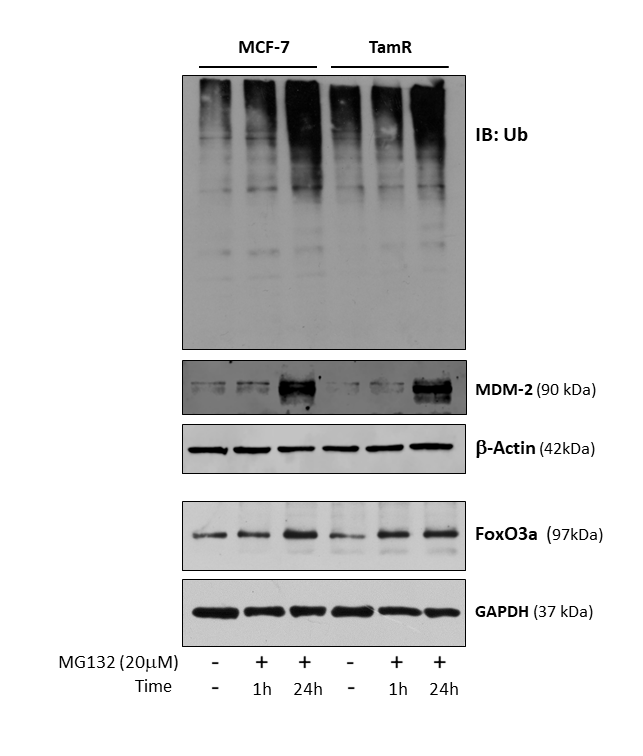


**Figure S4.** MG132 treatment inhibits FoxO3a and MDM2 protein degradation. MCF-7 and TamR cells were seeded in 60mm dishes, starved in PRF-SFM and treated with 20 µM MG132 for 1 h or 24 h or left untreated (−). Then cells were harvested and subjected to WB analysis. Antibodies against ubiquitin (Ub), FoxO3a and MDM2 were used to detect the relative proteins. MG132 treatment for 24h causes a general increase of the cellular ubiquitination process leading to a dramatic accumulation of MDM2 in both cell lines, while FoxO3a was affected as soon as after 1h of MG132 treatment (see also “Original blots and densitometries”). β-Actin and GAPDH were used as a loading control.


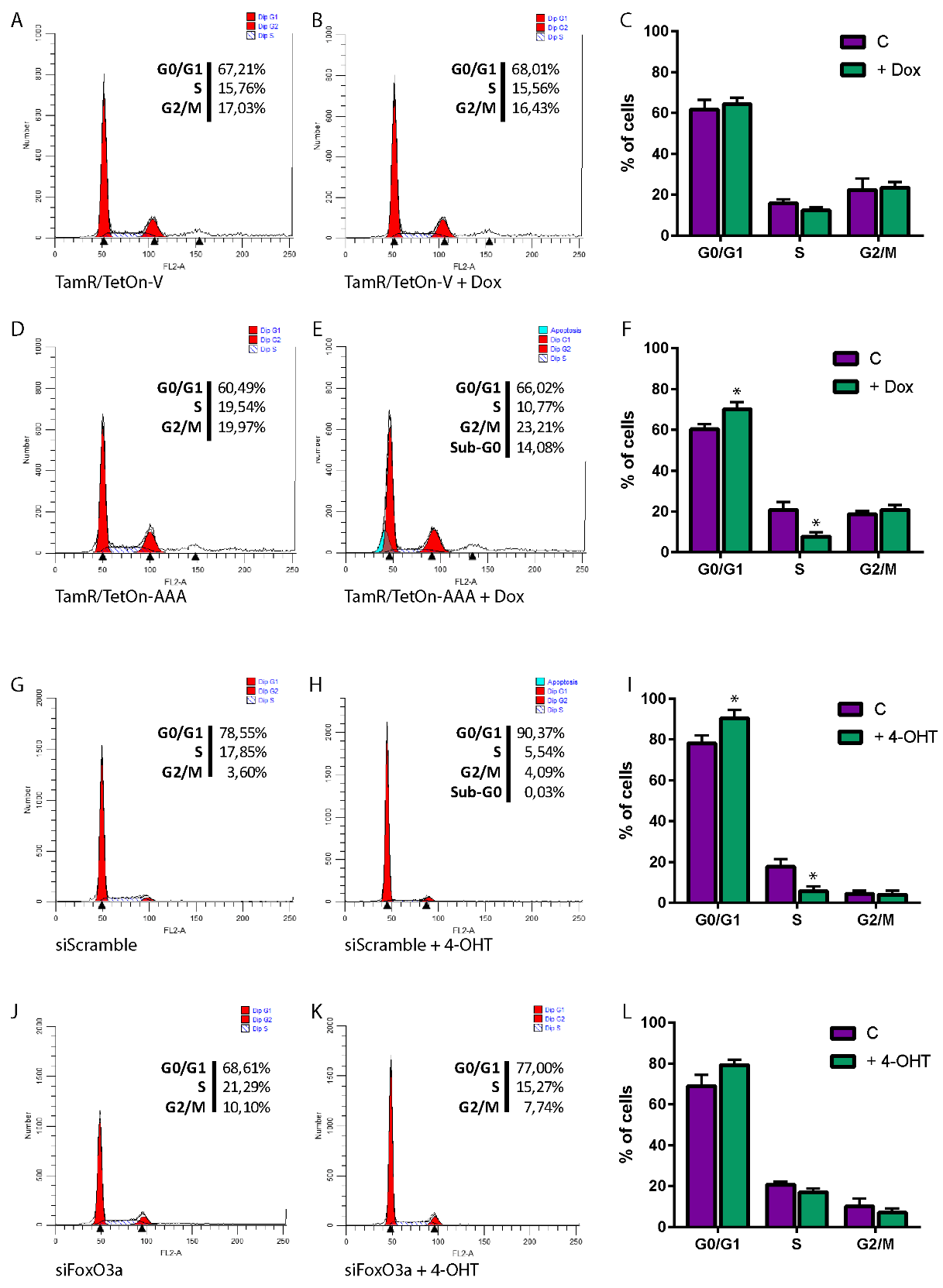


**Figure S5.** FoxO3a decreases the proliferation of TamR breast cancer cells. TamR/TetOn-V (**A–C**) and TamR/TetOn-AAA (**D–F**) cells were treated or not with Dox (3 µg/mL). MCF-7 cells were transfected with siScrambled (**G–I**) and with siRNA for FoxO3a (**J–L**) as described in *Materials and Methods* and treated or not with 4-OHT (1 μM). After 48 h cells were subjected to cell cycle analysis (see *Materials and Methods*). The percentage of cells in the G0/G1, S, and G2/M phases of the cell cycle are reported. The graphs derive from one original experiment, while the histograms on the right side represent the results that are expressed as mean from three independent experiments.







B

A







D

C

**Figure S6.** TEM observations on Dox-treated TamR/TetOn-AAA superrnatants. (**A**,**C**) Representative micrographs (1500× magnification) showing apoptotic cells that appear highly condensed with very large vacuoles and dark cytoplasm. Nuclear material undergoes condensation forming distinct masses of chromatin seen as small compact patches throughout the nucleus. At higher magnification (5000×) (**B,D**), the nuclear membrane appears highly crenated. The nucleus contains highly electron-dense chromatin mass. The cytoplasm is extensively altered, with marked accumulation of vacuoles of varying sizes.

**
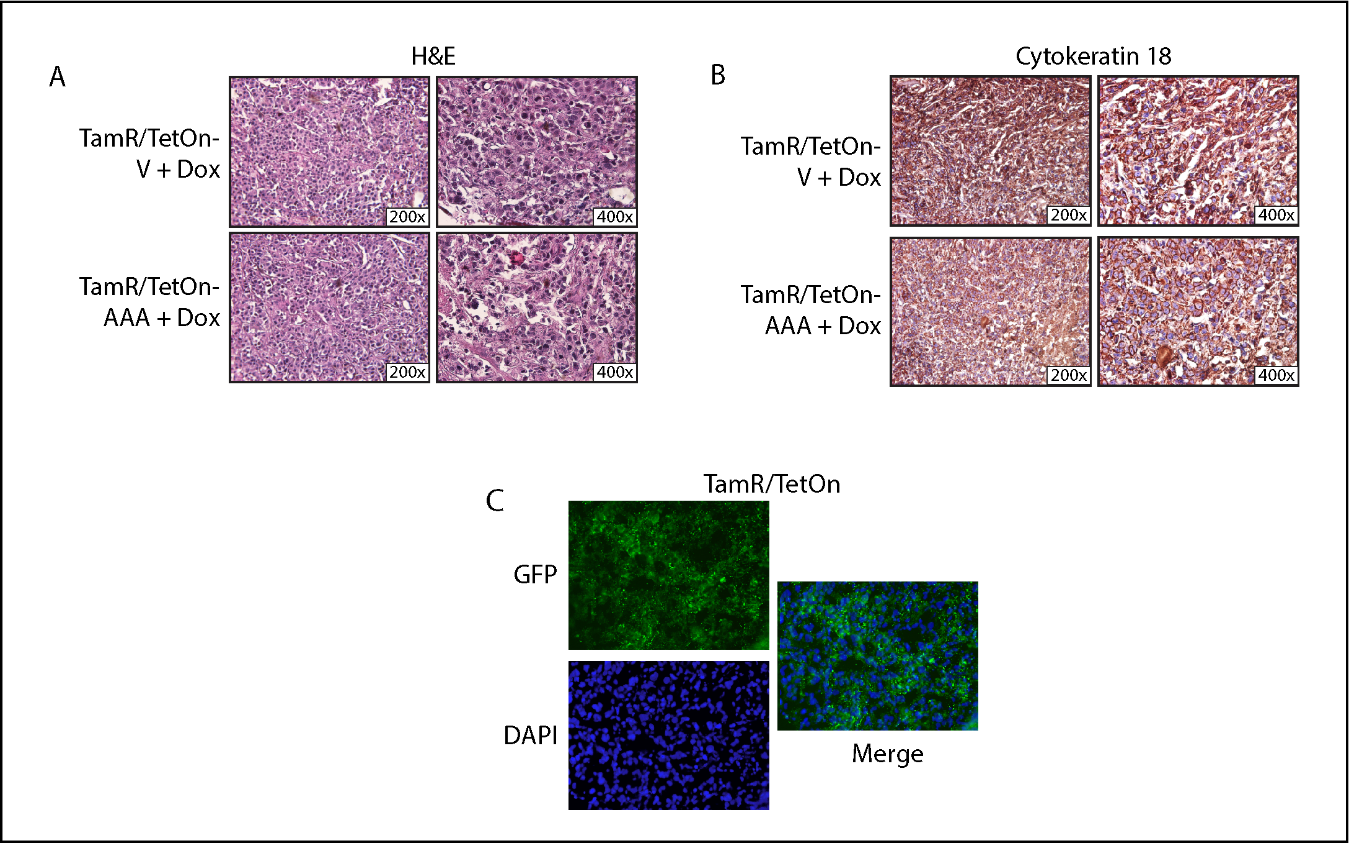
**

**Figure S7.** Histologic analysis of TamR/TetOn derived tumor xenografts. (**A**) FFPE sections of tumor xenografts stained with Hematoxylin and eosin (H&E) evidenced their composition in tumor epithelial cells. (**B**) The epithelial nature of the tumors was confirmed by immunostaining for the human epithelial cell marker cytokeratin 18 (Cyt18). (**C**) The strong GFP signal observed in xenograft sections deriving from TamR/TetOn cells, constitutively expressing GFP, due to the EGFP cassette contained in the pTRE-vector (see “*Generation of tamoxifen resistant cell lines*” in Supplementary Methods).


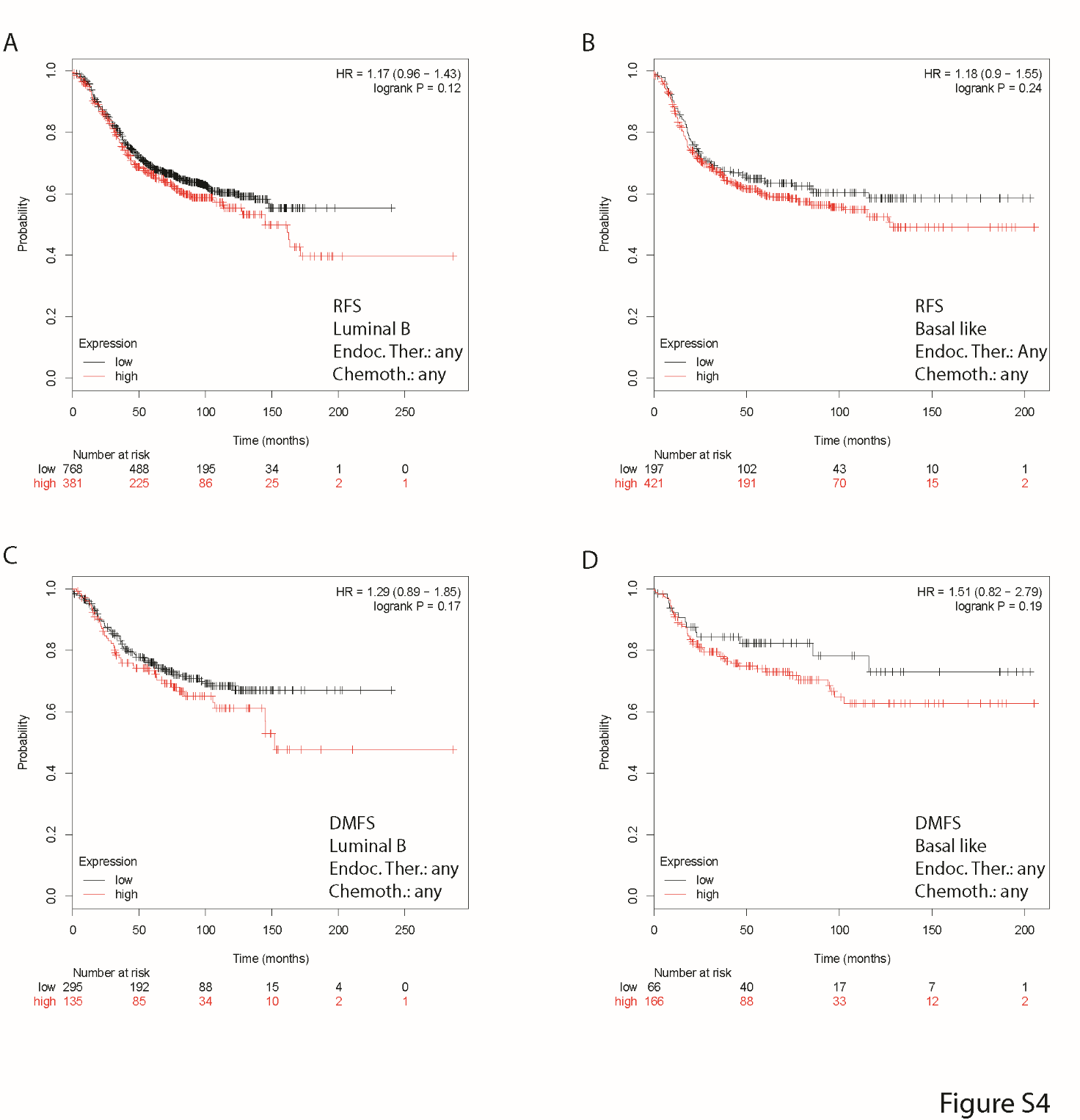


**Figure S8.** Kaplan-Meier analysis: FoxO3a loses its positive prognostic value in Luminal B and Basal-like BC patients. Relapse-free survival (RFS) and Distant metastasis free survival (DMFS) were calculated using microarray data from luminal B [RFS 1149 patients (**A**) and DMFS 430 patients (**C**)] and Basal-like [RFS 618 patients (**B**) and DMFS 232 patients (**D**)] subtype cohorts of patients. Kaplan-Meier (K-M) correlations were plotted for above median (high, in red) and below median (low, in black) FoxO3a gene expression. Details are reported in *Methods*.


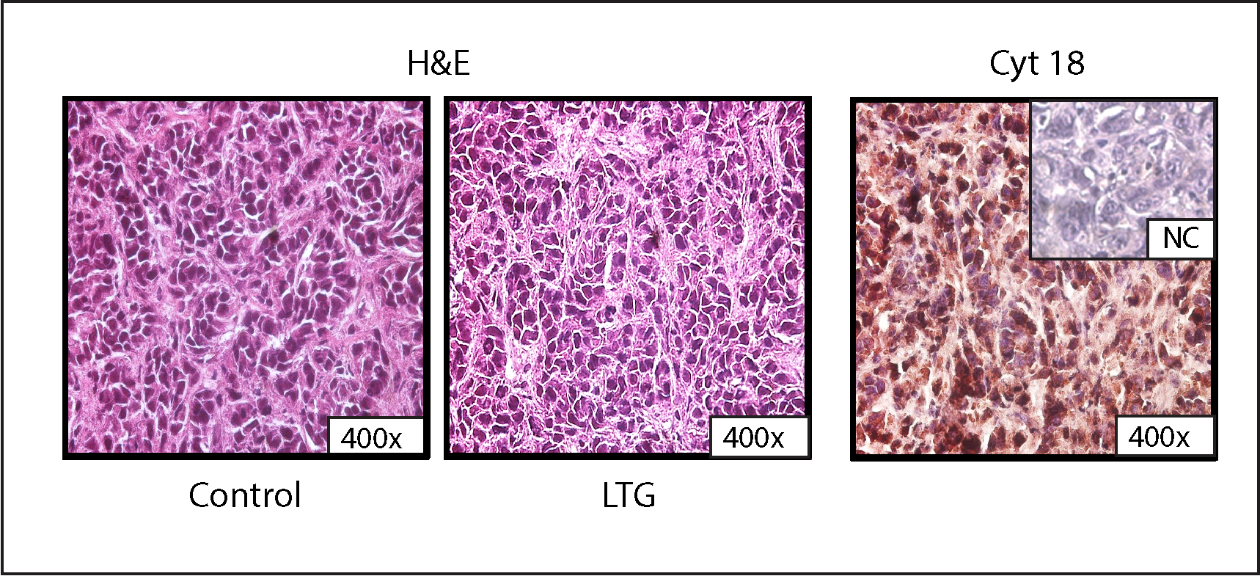


**Figure S9.** Histologic analysis of TamR derived tumor xenografts treated or not with LTG. H&E as well as Cyt18 staining showed that xenografts were primarily composed of tumor epithelial cells. Representative tumor sections from mice at 28 days were formalin fixed, paraffin embedded, sectioned, and stained with hematoxylin and eosin Y (H&E) or incubated with antibodies directed against the epithelial marker cytokeratin 18 (Cyt 18). The insets in Cy18 are representative images of negative control sections, where the primary antibody was replaced by nonimmune serum.
