# Supplementary material for: FoxO3a as a Positive Prognostic Marker and a Therapeutic Target in Tamoxifen-Resistant Breast Cancer
Source: Cancers (Basel). 2019 Nov 25;11(12):1858. doi: 10.3390/cancers11121858 (PMC6966564; doi:10.3390/cancers11121858)
Supplement: Supplementary file 1 [file cancers-11-01858-s001.zip › Original blots and densitometry.pdf]

FIGURE 1

Panel B

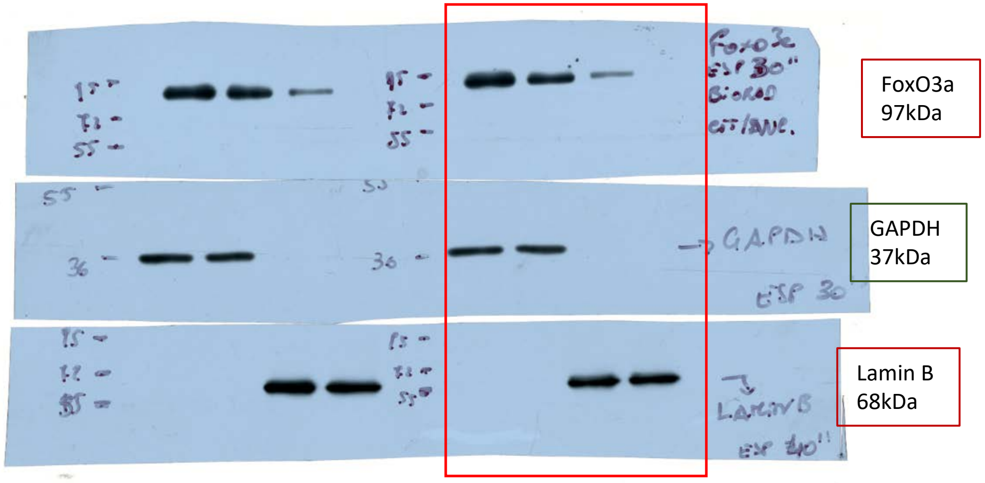

Lane	1	2	3	4
Foxo3a	23	15	7	0
GAPDH	15	13		
Lamin B			23	20
Ratio	1,5	1,1	0,3	0

FIGURE 1 Panel D

72
55
55
36

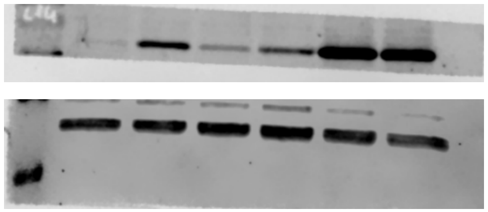

pAkt 60kDa

ACTIN 42kDa

Lane	1	2	3	4	5	6
pAkt	1	22	6	13	41	38
Actin	22	24	26	30	24	22
Ratio	0,04	0,91	0,23	0,43	1,70	1,72

Lane	1	2	3	4	5	6
pAkt	0,04	0,91	0,23	0,43	1,70	1,72
Akt	1,05	1,21	0,86	0,98	1,07	0,98
Ratio	0,04	0,75	0,27	0,44	1,59	1,76

72
55
55
36

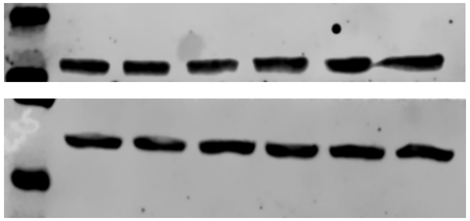

Akt 60kDa

ACTIN 42kDa

Lane	1	2	3	4	5	6
Akt	40	41	38	43	46	43
Actin	38	34	44	44	43	44
Ratio	1,05	1,21	0,86	0,98	1,07	0,98

250
130
95
55
36

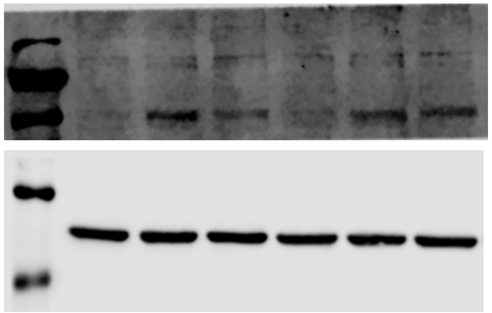

pFoxO3a (Akt-dip) 97kDa

ACTIN 42kDa

This Actin was used in figure as representative of each Actin evaluated

Lane	1	2	3	4	5	6
pFoxO3a	2	23	9	5	20	21
Actin	28	31	28	26	27	29
Ratio	0,07	0,74	0,32	0,19	0,74	0,72

Lane	1	2	3	4	5	6
pFoxO3a	0,07	0,74	0,32	0,19	0,74	0,72
FoxO3a	0,55	0,48	0,37	0,12	0,21	0,17
Ratio	0,13	1,54	0,86	1,58	3,52	4,24

130
95
72
55
36

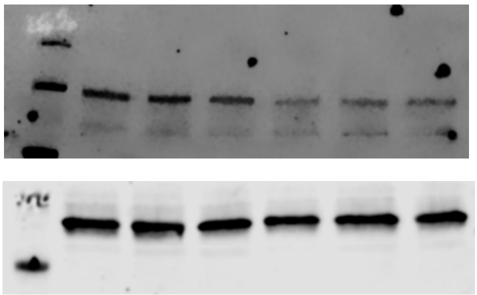

FoxO3a 97kDa

ACTIN 42kDa

Lane	1	2	3	4	5	6
Foxo3a	16	14	11	3	6	5
Actin	29	29	30	26	29	30
Ratio	0,55	0,48	0,37	0,12	0,21	0,17

FIGURE 1 Panel E

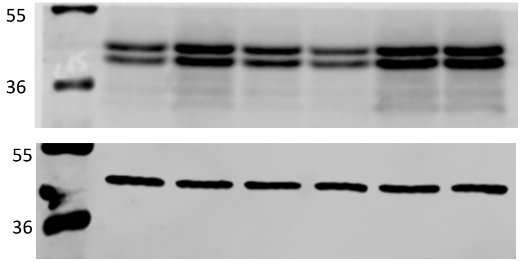

pMAPK 42-44kDa

ACTIN 42kDa

This Actin was used in figure as representative of each Actin evaluated

Lane	1	2	3	4	5	6
pMAPK	14	28	17	11	30	29
Actin	14	12	12	14	13	14
Ratio	0,8	2,3	1,4	0,8	2,3	2,1

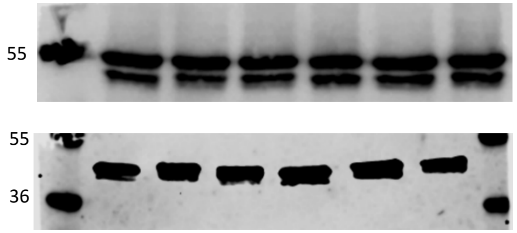

MAPK 42-44kDa

ACTIN 42kDa

Lane	1	2	3	4	5	6
MAPK	28	29	27	28	30	28
Actin	19	20	20	21	20	20
Ratio	1,5	1,4	1,3	1,3	1,5	1,4

Lane	1	2	3	4	5	6
Ratio pMAPK	0,8	2,3	1,4	0,8	2,3	2,1
Ratio MAPK	1,5	1,4	1,3	1,3	1,5	1,4
Ratio	0,5	1,6	1,0	0,6	1,5	1,5

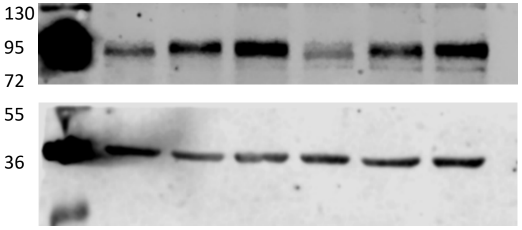

pFoxO3a (MAP-dip) 97kDa

GAPDH 37kDa

Lane	1	2	3	4	5	6
pFoxO3a	14	22	29	8	20	27
Actin	17	14	15	15	14	14
Ratio	0,8	1,6	1,9	0,5	1,4	1,9

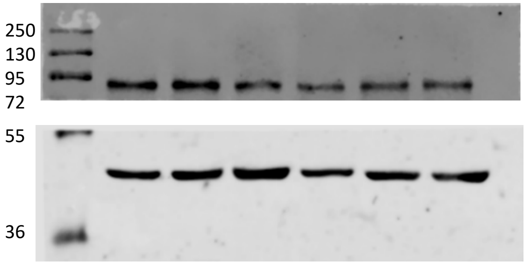

FoxO3a 97kDa

ACTIN 42kDa

Lane	1	2	3	4	5	6
FoxO3a	18	20	12	10	12	11
Actin	15	15	17	14	15	16
Ratio	1,2	1,3	0,7	0,7	0,8	0,7

Lane	1	2	3	4	5	6
Ratio pFoxO3a	0,8	1,6	1,9	0,5	1,4	1,9
Ratio FoxO3a	1,2	1,3	0,7	0,7	0,8	0,7
Ratio	0,7	1,2	2,7	0,7	1,8	2,7

FIGURE 1 Panel G

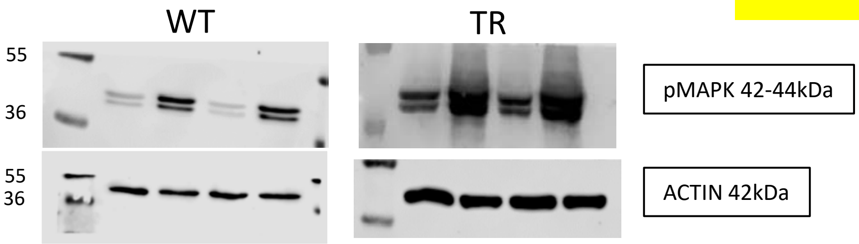

This Actin was used in figure as representative of each Actin evaluated

Lane	1	2	3	4		5	6	7	8
pMAPK	5	26	3	37		41	123	40	117
Actin	21	17	18	17		26	24	26	25
Ratio	0,2	1,5	0,2	2,1		1,6	5,1	1,5	4,7

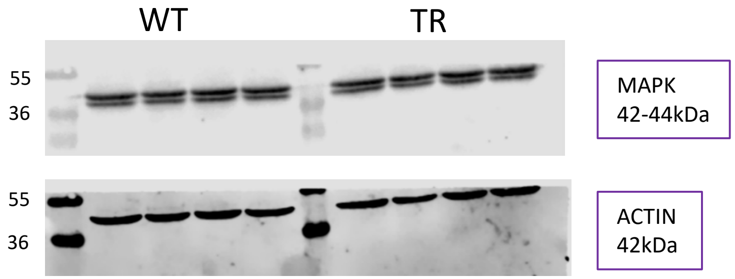

Lane	1	2	3	4		5	6	7	8
MAPK	29	29	31	30		26	24	28	30
Actin	20	20	22	21		17	15	17	16
Ratio	1,4	1,4	1,4	1,4		1,5	1,6	1,6	1,8

Lane	1	2	3	4		5	6	7	8
Ratio pMAP	0,2	1,5	0,2	2,1		1,6	5,1	1,5	4,7
Ratio MAP	1,4	1,4	1,4	1,4		1,5	1,6	1,6	1,8
Ratio	0,1	1,1	0,1	1,5		1,1	3,2	0,9	2,6

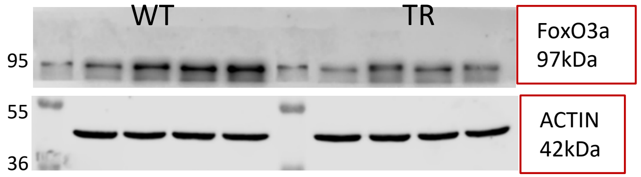

Lane	1	2	3	4		5	6	7	8
FoxO3a	10	14	16	20		7	10	9	9
Actin	22	20	21	20		22	20	20	20
Ratio	0,5	0,7	0,8	1,0		0,3	0,5	0,5	0,5

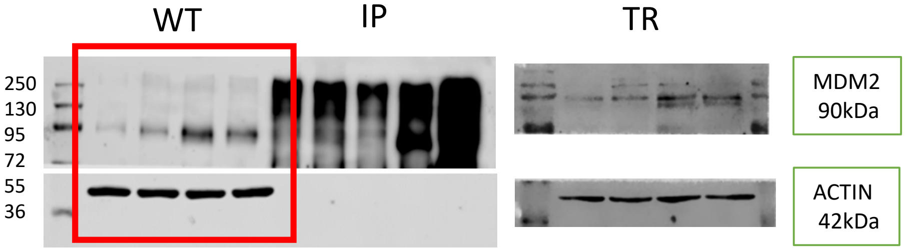

This Actin was used in figure as representative of each Actin evaluated

Lane	1	2	3	4		5	6	7	8
MDM2	3	7	16	14		6	8	14	13
Actin	21	18	20	17		12	11	11	12
Ratio	0,1	0,4	0,8	0,8		0,5	0,7	1,3	1,1

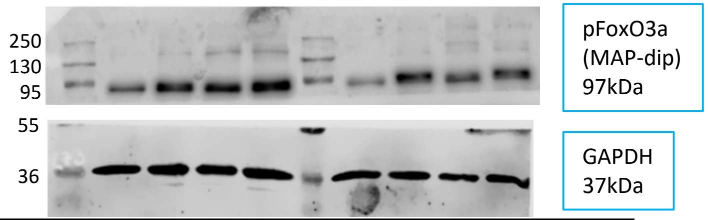

Lane	1	2	3	4		5	6	7	8
pFoxO3a	15	27	29	33		8	27	18	23
Actin	28	31	29	31		21	22	16	16
Ratio	0,5	0,9	1	1,1		0,4	1,2	1,1	1,5

FIGURE 2

Panel B

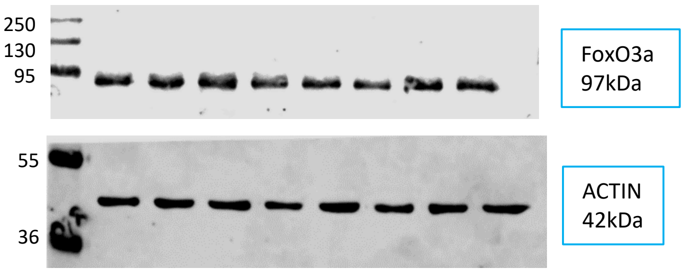

Lane	1	2	3	4	5	6	7	8
FoxO3a	15	17	25	22	14	13	18	16
Actin	22	23	23	18	24	20	22	23
Ratio	0,7	0,7	1,1	1,2	0,6	0,7	0,8	0,7

Panel D

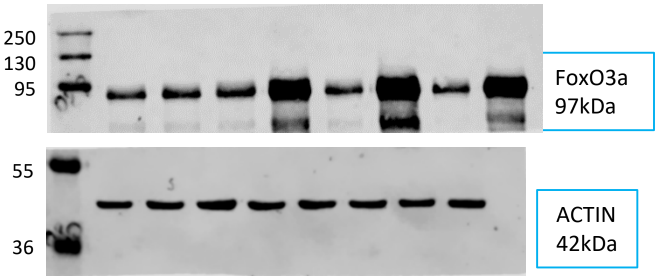

Lane	1	2	3	4	5	6	7	8
FoxO3a	10	11	9	24	11	32	10	27
Actin	9	10	14	11	11	10	9	11
Ratio	1,1	1,1	0,6	2,2	1,0	3,2	1,1	2,5

Panel C

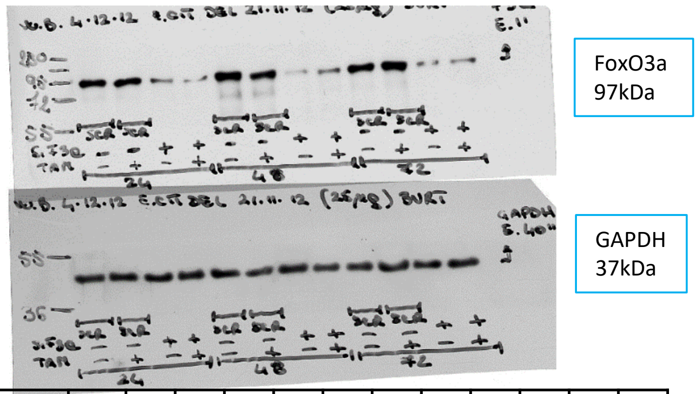

Lane	1	2	3	4	5	6	7	8	9	10	11	12
FoxO3a	25	26	12	12	27	28	14	13	26	27	11	15
Actin	27	26	26	26	27	25	25	29	29	25	27	26
Ratio	0,9	1,0	0,5	0,5	1,0	1,1	0,6	0,4	0,9	1,1	0,4	0,6

FIGURE 2

Panel I

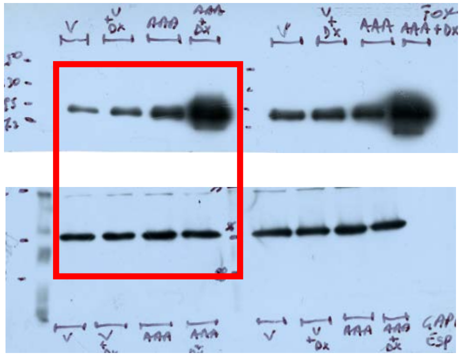

FoxO3a 97kDa

GAPDH 37kDa

Lane	1	2	3	4
Foxo3a	8	13	105	787
GAPDH	17	18	22	20
Ratio	0,5	0,7	4,8	39

Panel K

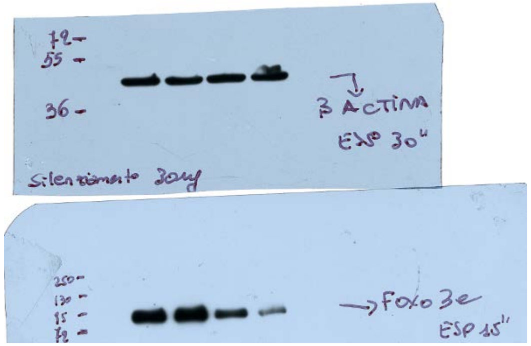

Lane	1	2	3	4
Foxo3a	30	52	16	8
Actin	18	17	19	22
Ratio	1,2	1,1	0	0

FIGURE 2

Panel O

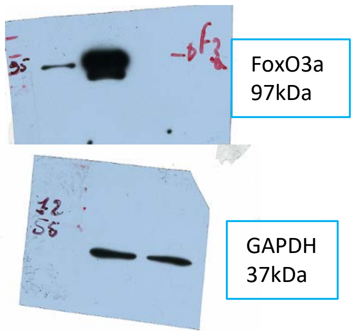

Lane	1	2
Foxo3a	6	81
GAPDH	8	13
Ratio	0,7	6,2

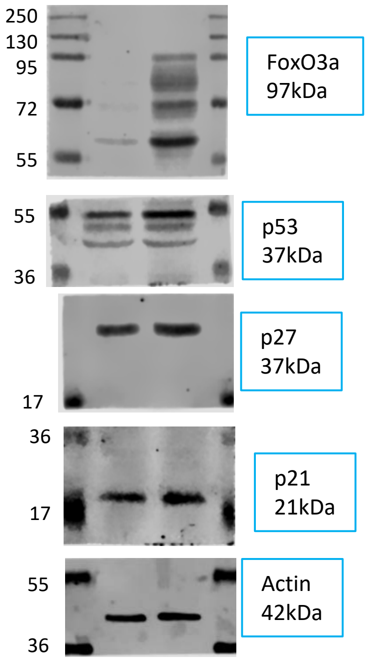

Lane	1	2
FoxO3a	0,9	26
p53	43	66
p27	43	62
p21	47	75
Actin	79	82

Panel P

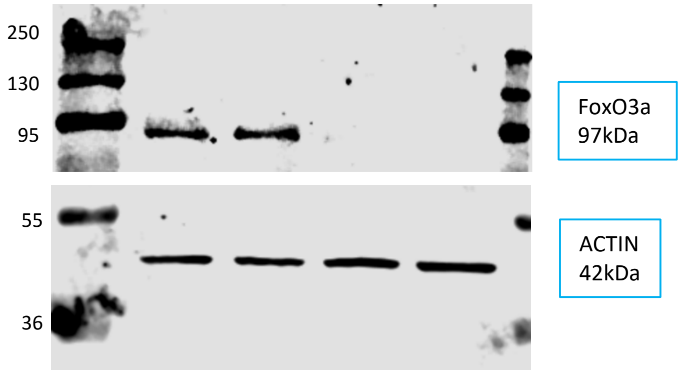

Lane	1	2	3	4
Foxo3a	19	17	0	0
Actin	16	15	19	19
Ratio	1,2	1,1	0	0

FIGURE 4

Panel E

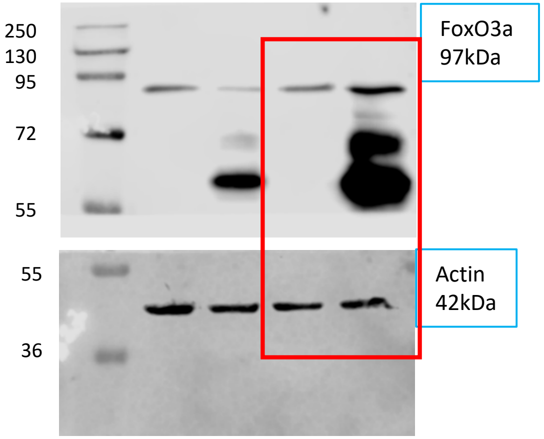

Lane	1	2
Foxo3a	17	30
Actin	34	29
Ratio	0,5	1,0

Panel F

Lane	1	2
P53	19	37
P27	19	30
p21	15	25
Actin	15	16
Ratio p53	1,2	2,3
Ratio p27	1,2	1,8
Ratio p21	1	1,6

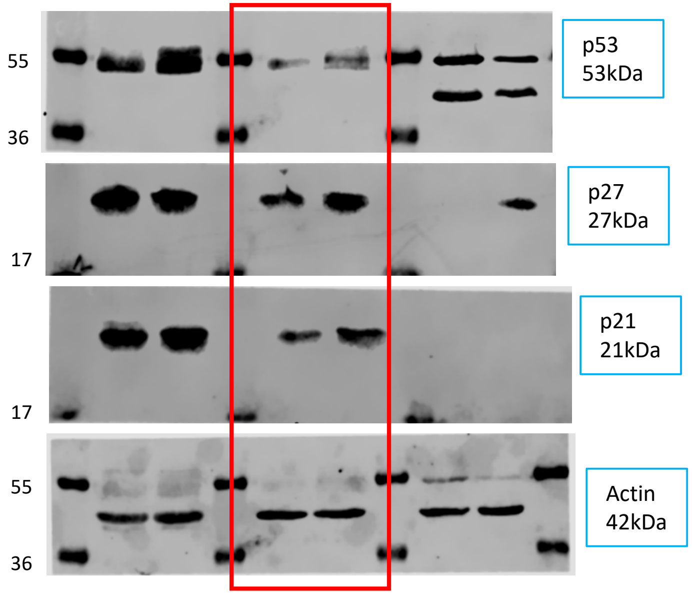

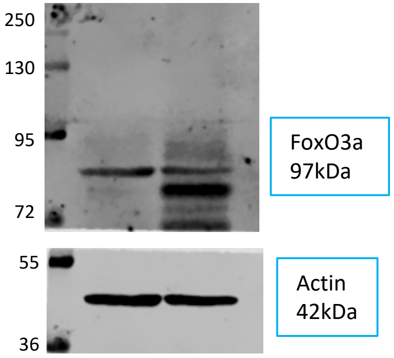

Lane	1	2
Foxo3a	14	38
Actin	35	32
Ratio	0,4	1,2

FIGURE 6

Panel F

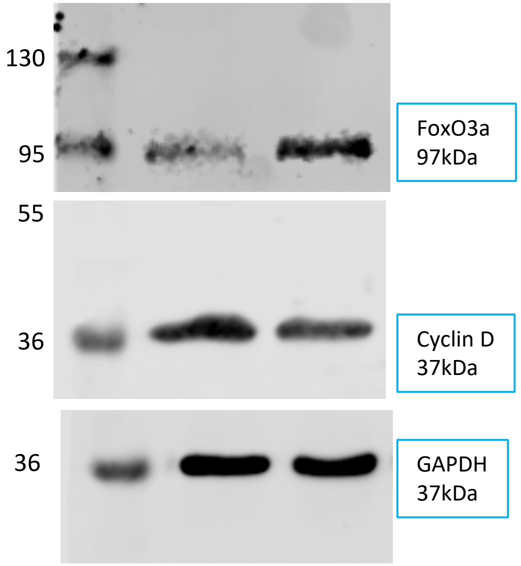

Lane	1	2
Foxo3a	25	52
Cyclin D	70	40
GAPDH	70	66
Ratio Fox	0,4	0,8
Ratio Cyc	1	0,6

FIGURE S1

Panel C

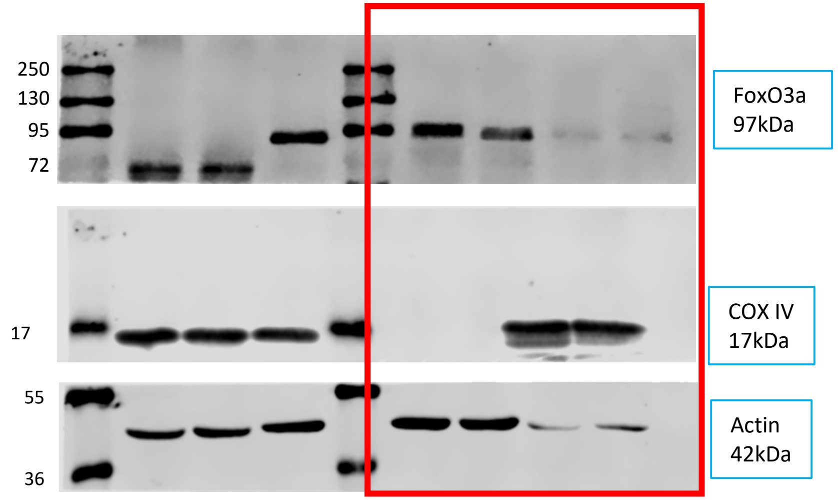

Lane	1	2	3	4
Foxo3a	17	10	1,2	1,3
COX IV	0	0	22	19
Actin	19	17	2,1	4,4
Ratio	0,9	0,6	0,05	0,07

FIGURE S2

Panel A   Panel D

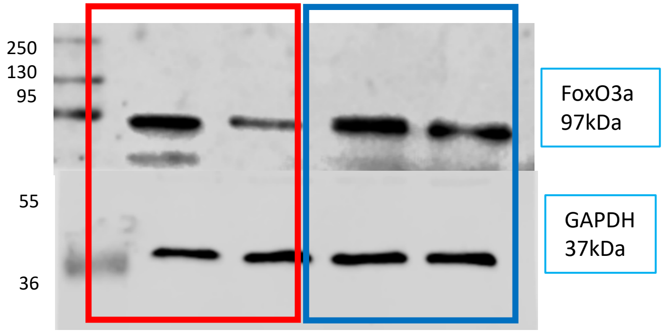

Lane	1	2	3	4
Foxo3a	58	18	57	28
GAPDH	19	23	23	23
Ratio	3,1	0,7	2,5	1,1

Panel C

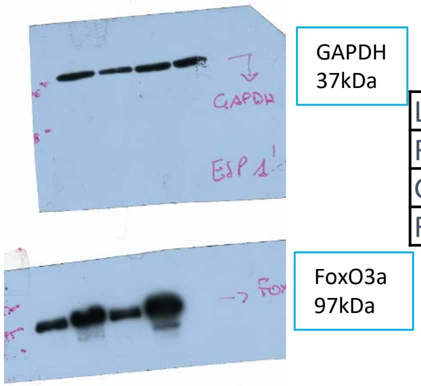

Lane	1	2	3	4
Foxo3a	20	38	20	54
GAPDH	16	10	17	16
Ratio	1,3	3,8	1,2	3,4

Panel F

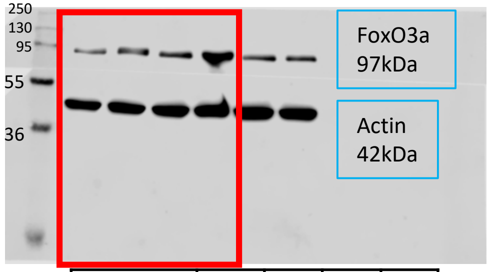

Lane	1	2	3	4
Foxo3a	21	39	57	92
Actin	62	56	59	68
Ratio	0,34	0,7	0,97	1,35

FIGURE S4

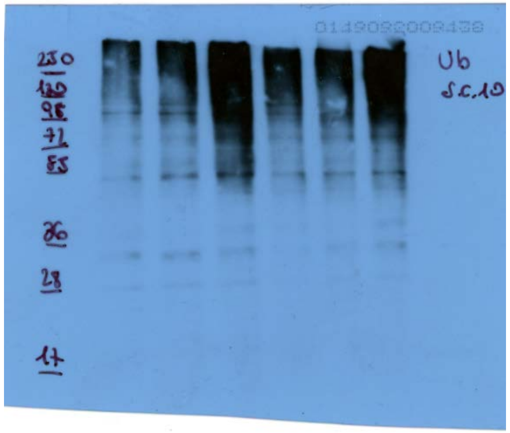

IB: Ub
97kDa

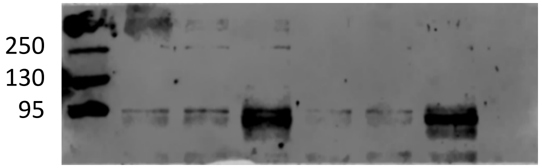

MDM-2
90kDa

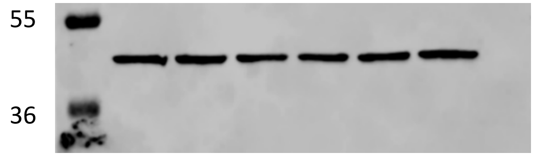

Actin
42kDa

Lane	1	2	3	4	5	6
Ub	22	43	89	52	56	77
MDM-2	1,3	2,3	12	0,4	1,1	12
Actin	16	17	15	16	16	17
Ratio Ub	1,4	2,5	5,9	3,2	3,5	4,5
Ratio MDM-2	0,2	0,1	0,8	0,02	0,06	0,7

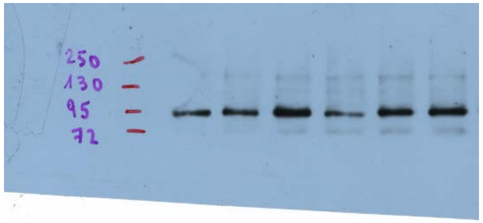

FoxO3a
97kDa

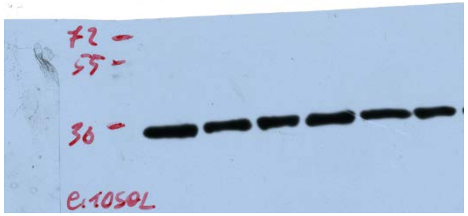

GAPDH
37kDa

Lane	1	2	3	4	5	6
FoxO3a	8,7	10	19,7	6,8	13	15
GAPDH	25	17	22	26	20	20
Ratio	0,3	0,6	0,9	0,2	0,6	0,7
